# Cell Carriers for Oncolytic Virus Delivery: Prospects for Systemic Administration

**DOI:** 10.3390/cancers17142296

**Published:** 2025-07-10

**Authors:** Viktoria A. Sarkisova, Alexandra A. Dalina, Daria O. Neymysheva, Martin A. Zenov, Galina V. Ilyinskaya, Peter M. Chumakov

**Affiliations:** 1 Engelhardt Institute of Molecular Biology, Russian Academy of Sciences, 119991 Moscow, Russia; v.sarkisova@eimb.ru (V.A.S.); alexandra.dalina@gmail.com (A.A.D.); neymyshevadaria@gmail.com (D.O.N.); martin.zenov@yandex.ru (M.A.Z.); ilyinskaya@yahoo.com (G.V.I.); 2 Department of Virology, Faculty of Biology, Lomonosov Moscow State University, 119991 Moscow, Russia

**Keywords:** oncolytic virus, virotherapy, cell therapy, cell carriers, systemic virus delivery

## Abstract

Treatment of malignant diseases using oncolytic viruses (OVs) is currently considered a promising therapeutic approach. Initial encouraging results fueled a large number of clinical trials, showcasing favorable safety profiles of OVs—but therapeutic outcomes remain far from perfect. The efficacy of systemically administered OVs is limited due to rapid immune clearance and suboptimal biodistribution, while locally administered OVs encounter an additional barrier of poor bioavailability. Cell-based carriers that can shield viral particles and provide tumor-targeted OV delivery, represent one of the potential ways to address these challenges. The feasibility of this approach was demonstrated using a broad range of cell types, including mesenchymal stem cells (MSCs), neural stem cells (NSCs), different subsets of immune cells, and cancer cell lines. The resulting spectrum of carriers can be viewed as a multifaceted tool, taking into account the specific properties, advantages, and limitations of each cell carrier type discussed in this review. Careful consideration of these features will provide the basis for successful development of cell-based OV delivery platforms.

## Simple Summary

Cell carriers represent a promising strategy to overcome barriers associated with systemic administration of oncolytic viruses, providing both protection from neutralizing factors and targeted delivery to tumors. Despite numerous proof-of-concept studies, the overall versatility of the approach remains unclear due to diverse cell types utilized in these studies, as well as different experimental conditions. In this review, we aimed to summarize performance and key features of known cell carrier types, paying special attention to specific models and administration routes. On the basis of this analysis, we also provide potential suggestions for the optimization of cell-based virus delivery.

## 1. Introduction

Virotherapy is emerging as a unique cancer treatment, combining selective lysis of cancer cells with induction of antitumor immunity. Oncolytic activity is an intrinsic feature of viruses from different families, including RNA-containing mammalian orthoreovirus type 3 Dearing (ReoT3D), Newcastle disease virus (NDV), vesicular stomatitis virus (VSV), DNA-containing vaccinia virus (VV), herpes simplex virus 1 (HSV-1), and oncolytic adenoviruses (oAds), among others [1,2,3,4]. Attenuated vaccine strains of pathogenic viruses have served as platforms for developing safe modified variants with enhanced tumor tropism and immunomodulatory properties [5,6,7]. Genetic modifications enable the creation of tailored OV strains targeting specific cancer types, although the extent of these modifications is limited by the genomic characteristics of the parental virus. HSV-1 and adenoviruses are frequently used as backbones due to their large, stable genomes, which are easily engineered for safety and therapeutic gene delivery. The direct tumoricidal mechanisms of OVs generally do not overlap with those of chemotherapy or targeted therapies. OVs can eliminate cancer cells that are resistant to apoptosis and can destroy cancer stem cells, which are typically refractory to conventional therapies [8,9,10]. Currently, OVs are largely investigated as potential auxiliary agents in combination with other treatment regimens including immuno-, chemo-, and targeted therapies [11,12,13]. The ability of OVs to induce immunogenic cell death and prime NK- and T-cell-mediated antitumor responses provides a rationale for combining virotherapy with immune checkpoint blockade. Notably, OV infection has been shown to upregulate PD-L1 levels at tumor sites via type I interferon signaling [14,15], suggesting that PD-L1 blockade could mitigate this unwanted immunosuppressive response.

Clinical investigations revealed that OVs were generally well-tolerated at high doses and had favorable safety profiles [1,16]. Despite these unique advantages, many OVs that were evaluated in the later stages of clinical trials, failed to reach the endpoint as a monotherapy [17,18,19]. The only FDA-approved OV drug to date is Talimogene laherparepvec (T-VEC), a genetically modified HSV-1 encoding granulocyte-macrophage colony-stimulating factor (GM-CSF). T-VEC is approved for the local treatment of melanoma that has recurred after initial surgery and spread to the skin, soft tissue, or lymph nodes (LNs) [20]. Viewing T-VEC as the current “gold standard” of virotherapy, no OVs with alternative routes of administration have yet received any kind of FDA approval.

Intratumoral (IT) administration is used in most current OV clinical trials [21,22]. Recently, FDA accepted the biologics license application for another oncolytic agent RP1 in combination with nivolumab for the treatment of advanced, anti-PD-1 failed melanoma. RP1, or vusolimogene oderparepvec, represents HSV-1 equipped with fusogenic protein and GM-CSF and is given by IT injection similarly to T-VEC [23]. Unsurprisingly, in the case of conditionally and non-replicating viral vectors that are essentially more limited in their spread, locoregional inoculation similarly serves as a key promising route in some indications. As an example, intravesical injection was utilized in a phase 3 clinical trial featuring adenovirus-based Cretostimogene grenadenorepvec, resulting in 75.2% complete response rate in high-risk, BCG-unresponsive non-muscle invasive bladder cancer [24]. These results illustrate that in the absence of delivery barriers, both vector- and OV-based therapies are able to produce impressive late-stage clinical trial outcomes.

**Figure 1:**
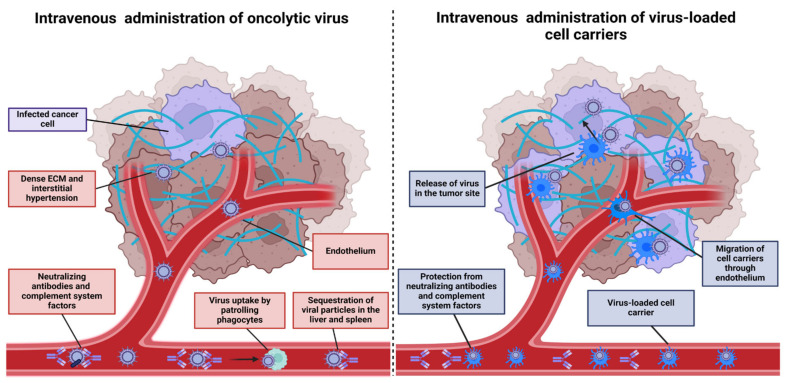
Common factors limiting the spread of IV-administered OV particles include rapid clearance from the bloodstream and physical barriers within the tumor microenvironment. OV-loaded cell carriers can partially overcome these obstacles, improving OV penetration into tumors.

Unfortunately, only a small proportion of tumors are accessible for direct injection; most cases involve solid tumors or metastases in internal organs. However, several obstacles arise shortly after OV injection (Figure 1).

As demonstrated by Willmon et al., within just two minutes after IV injection of VSV in immunocompetent mice, the vast majority of virus particles were associated with immune cells, indicating a short-lived presence of free virions in the bloodstream [25]. The fate of these cell-associated virions remains unclear; while some may be degraded, it is possible that such “packaging” provides partial protection from neutralizing antibodies.

Possibly, long-term existence of the infectious particles in the bloodstream appears uncommon for non-bloodborne viruses. For instance, human immunodeficiency virus (HIV) has remarkable capability to reach CD4+ T-cells in the LNs using DC-SIGN+ dendritic cells as naturally occurring carriers. In contrast, OVs that employ other transmission routes lack evolutionary adaptations to retain in circulation for prolonged time [26,27].

Antibodies and the complement system, which in turn facilitate the neutralization and rapid sequestration of OVs by patrolling phagocytic immune cells, remain a crucial limiting factor for efficient OV spread [28]. In the CT26-bearing mice model, IV infused VSV-GFP was able to reach tumors only in naive animals. Immunization or prior exposure to VSV several months earlier completely abolished VSV replication [29]. Repeated OV injections during treatment inevitably amplify antiviral humoral responses, further impeding therapeutic efficacy. Additionally, high seroprevalence has been reported for several OVs circulating in the human population or used as vaccine vectors, including vaccinia virus (VV), reoviruses, and adenovirus type 5 [30].

Physical barriers represent an additional problem for OV spread, both in the case of IT and IV administration. Dense stromal tissue is an essential component of many solid tumors that confers chemo and immunotherapy resistance and lowers the chance of virus reaching target cells, especially in the case of OVs that were modified to infect only malignant cells. Abnormal microvascular architecture and vessel compression caused by proliferating cancer cells result in interstitial hypertension and reduced blood supply, confining OV spread primarily to regions near blood vessels [31,32].

Inhibition of the antiviral immune response by immunosuppressive drugs was investigated as one of the potential ways to prolong and enhance OV replication. Cyclophosphamide (CPA) combined with OVs has shown promise in several studies; however, disentangling the pleiotropic antitumor effects of CPA from its immunomodulatory effects remains challenging [33,34,35,36]. Conversely, transient immunosuppression using agents such as tacrolimus, mycophenolate mofetil, or methylprednisolone sodium succinate failed to significantly improve OV retention in mouse models [37]. Notably, replication efficiency of OVs does not always correlate with therapeutic outcome—in some cases, tumor elimination is a result of solely immune-mediated clearance [38]. Moreover, antiviral immunity has the ability to boost antitumor response, as shown for NDV [39].

Dose escalation of systemically administered OVs intuitively seems to be another potential way to overcome the limitations described above. However, this approach is associated with higher treatment costs and an increased risk of side effects [40]. An alternative solution is to shield OV particles using nanomaterials, capsid modifications, or cell-based carriers [41]. Beyond providing virus protection, some types of cell carriers are capable of supporting OV replication and migrating to tumor sites, resulting in significant improvements in delivered dose and biodistribution compared to other strategies.

## 2. What Makes a Perfect Cell Carrier?

Willmon et al. elegantly compared cell-based OV carriers to “FedEx couriers,” highlighting their role in protecting and delivering the viral “package” to tumor sites [25]. The primary goal of these “cell couriers” is to ensure that the virus reaches its target while being shielded from neutralizing factors. The ideal IV-administered cell carrier should reversibly bind OVs, circulate freely in the bloodstream, and protect the virus from neutralizing factors. The mode of interaction—surface adsorption or internalization—determines the delivery mechanism.

In the case of surface-bound OVs, transfer occurs through direct contact with the target cell. Unexpectedly, such delivery mode was found to be effective even in the presence of neutralizing antibodies, but only when low doses of virus were used for loading. This phenomenon was demonstrated for ReoT3D- and VSV-loaded T-cells in immunized murine models [42,43]. For T-cells loaded with measles virus (MV), virus transfer was completely abolished in mice passively immunized with high doses of serum but was able to occur when lower doses of serum were administered [44]. These results suggest that a specific threshold level of neutralizing antibodies determines the success rate of surface-bound virus delivery. Additionally, the kinetics and frequency of particle shedding from the cell carrier membrane remain unclear in vivo, suggesting that some amount of virus may be released before the cell carrier reaches its target cells. Nevertheless, this mode of delivery can be used in an immune-naive setting.

Internalization of OV particles by cell carriers either with or without subsequent replication provides more efficient protection against neutralizing antibodies. A remarkable study by Ilett et al. compared different delivery modes using T-cells and bone marrow-derived dendritic cells (BMDCs). Mature BMDCs that were able to internalize ReoT3D significantly outperformed T-cells in clearing lymph node metastases in immunized mice, though one cannot exclude that this difference was also influenced by different chemotactic activity of these cell subsets [43]. Similarly, monocytes that were able to internalize antibody-reovirus complexes successfully delivered infectious particles to tumors despite lack of replication [45].

As with surface-bound transfer, it remains unknown whether the release of internalized viruses follows time-dependent patterns. Replication within cell carriers eventually increases the viral load delivered to tumors and is characterized by a predictable time of virus release. For OVs such as VSV, which have an extremely short replication cycle of approximately 6 h, it is crucial to ensure that cell carriers reach their destination before viral progeny are released. Tumor trafficking may take up to 72 h, as observed with T-cells [25].

Tumor-targeted migration is a positive feature inherent in many cell carrier types such as mesenchymal stem cells (MSCs) and various subsets of immune cells [46]. However, entrapment of cells within the microvasculature of the liver and lungs after IV infusion is common for large-sized carriers, thereby limiting migration efficiency. Finally, a substantial number of cell “couriers” is required to ensure successful delivery. Therefore, carriers must either be available for direct isolation from patient tissue samples in large quantities or be capable of rapid proliferation in culture.

## 3. Cell-Based Carriers for OV Delivery

A variety of cell types—including cancerous and non-cancerous lines, MSCs, NSCs, and immune cells—have been studied as OV carriers. Criteria for evaluation include delivery mode, OV compatibility, tumor tropism, and ease of isolation/expansion. While much emphasis has been placed on IV administration in this review, studies exploring alternative administration routes for cell carriers were also included, showcasing the overall potential of this approach to target tumors in different locations. Interestingly, OV-loaded carriers may prove beneficial even when injected directly into tumors, as they enhance virus retention compared to OV alone [47,48].

### 3.1. Mesenchymal Stem Cells (MSCs)

MSCs are multipotent stem cells present in adult organisms with the capacity to differentiate into various cell types of mesenchymal lineage. The concept of utilizing MSCs as carriers has gained significant attention in recent years and has been evaluated in multiple clinical trials, making this cell type probably the most studied one in the context of OV delivery [49,50].

MSCs can be efficiently expanded in culture and isolated from a variety of tissues, including bone marrow (BM) aspirate, adipose tissue (AT), dental pulp, endometrium, and umbilical cord blood (UCB). AT and BM are the most common sources for MSCs that are further expanded in vitro for therapeutic purposes, though AT is more preferential in terms of convenience [51]. Despite their different tissue origins, AT-MSCs and BM-MSCs acquire similar phenotypic and morphological characteristics under standard culture conditions [52,53]. The number of MSCs that can be isolated directly from tissue varies significantly among donors. In some cases, the expansion may take up to two weeks after isolation [54]. Although MSCs are generally considered genetically stable, long-term cultivation to obtain sufficient numbers for therapy has raised some safety concerns. Wang et al. described the spontaneous appearance of cell populations with abnormal karyotype in BM-MSC cultures, which were able to form tumors in NOD/SCID mice [55]. Another study showed that chromosomal aberrations could occur in BM-MSC cultures as early as the second or third passage [56]. However, a large number of clinical and preclinical studies in regenerative medicine support the overall safety of MSC-based therapy and report a low incidence of side effects [57].

The immunosuppressive properties of MSCs in the context of cancer therapy remain a matter of debate, even after decades of research [58]. MSCs are recognized as an important component of the tumor microenvironment and exert potent anti-inflammatory activity due to the diverse number of secreted molecules including IDO, PGE-2, and PD-L1 [59,60,61,62]. Notably, the inflammatory factors act as inducers of immunosuppressive secretome of MSCs [63]. Perhaps, the most remarkable demonstration of MSCs immunosuppressive capacity lies in exploiting MSC transplantation therapies for autoimmune disorders, along with recent FDA approval of allogeneic MSC-based therapy for steroid-refractory acute graft-versus-host disease [64].

Some OVs, such as oAds, eventually lyse MSCs several days after infection, suggesting that infected MSCs are unlikely to induce long-term immunosuppressive effects [65]. On the other hand, certain modifications of OVs may enhance the immunosuppressive activity of infected MSCs. For example, MSCs loaded with decorin-encoding adenovirus failed to induce an antitumor immune response compared to MSCs loaded with unmodified adenovirus [58].

MSC-based IV delivery has demonstrated promising results in targeting lung- and liver-localized cancerous lesions. UC-MSCs slightly supported the replication of decorin-expressing oAd (rAd.DCN) and facilitated the clearance of lung metastasis in 4T1 breast cancer murine model [66]. Another study has shown that BM-MSCs support replication of oncolytic oAd ICOVIR-5, and can be used to target A549 lung tumors in nude mice [67]. Similar results were demonstrated in the MDA-MB-231 pulmonary metastatic disease model for conditionally replicating adenovirus (CRAd) [68]. Myxoma virus (MxV)-loaded BM-MSCs induced regression of pulmonary melanoma lesions and increased survival of treated mice, compared to MxV alone [69]. In the models of ovarian cancer metastasis to lung in nude mice and breast cancer metastasis to brain in NSG mice, a single IV injection of MSCs that were forcedly infected with HER2-retargeted HSV reduced the metastatic burden in both settings. Notably, MSCs of different tissue origins had different susceptibility to HSV infection, and infection efficiency was enhanced in presence of polyethylene glycol [70].

In hepatocellular carcinoma (HCC), BM-MSCs loaded with HCC-targeted oAd inhibited growth of orthotopic HCC tumors more efficiently compared to virus alone. Notably, capsid modification was performed to support efficient uptake of virus by MSCs [65]. Earlier studies noted that MSCs express low levels of coxsackie and adenovirus receptor (CAR), which fueled the development of strategies to overcome CAR-dependence of adenoviral vectors [71,72]. In a subcutaneous model of pancreatic cancer, AsPC-1 xenografts in nude mice were successfully targeted by oAd/RLX-infected BM-MSCs. Forced infection of BM-MSCs was achieved through the uptake of oAd/RLX biodegradable polymer PCDP complex [73]. MSCs were shown to be permissive to MV infection and could serve as promising carriers in the orthotopic HCC model, transferring MV to cancer cells via heterofusion. Inhibition of the growth after MSC therapy was detected in both antibody-naïve and passively immunized SCID mice, where delivery of neat MV was completely inefficient [74].

Clinical evaluation of IV-infused, ICOVIR-5 loaded MSCs was conducted in a small cohort of children with metastatic neuroblastoma. Treatment was well-tolerated and one complete response was achieved [75]. Another clinical trial utilized autologous BM-MSCs as carriers of ICOVIR-5 in two cohorts of patients, including nine children and seven adults with various advanced malignancies. No severe adverse effects were observed, and adenoviral replication was detected in seven pediatric patients and in none of the adult cases. Two pediatric patients with neuroblastoma showed disease stabilization [50].

For brain tumors, intraarterial (IA) injection of loaded MSCs may offer greater efficiency. MSCs loaded with Delta24-RGD oAd significantly prolonged survival in mice with orthotopic glioma U87MG and U251 xenografts upon IA infusion [76]. Though being more preferable in terms of reaching the tumor site, the IA route is technically demanding and possesses substantial safety risks associated with cerebral embolism [77,78,79].

The elimination of melanoma brain metastases and prolonged survival in both immunocompetent and immunocompromised mouse models have been reported after intracranial administration of HSV-loaded MSCs, but not after HSV alone [80]. In a clinically relevant glioblastoma model, intracavitary injections of HSV-infected BM-MSCs were able to deliver the virus. IV-injected MSCs became trapped in the lungs and were unable to reach intracranial tumors [81].

Intraperitoneally (IP)-injected oAd5/3-MSCs delayed ovarian cancer growth more efficiently compared to IP-injected virus without carriers in SCID mice [82]. In a similar setting, IP-injected MV-infected MSCs localized to peritoneal tumors and were able to transfer virus in passively immunized athymic mice [83]. Comparing the IP versus IV administration of AT-MSCs in a pancreatic model revealed more pancreas-targeted distribution of the virus when AT-MSCs were delivered IP to mice bearing orthotopically injected Pan02 model. MxV-loaded AT-MSCs extended the survival of the treated animals and boosted expression of key adaptive immune response markers [84]. The IP route was also successfully applied in another study using MxV-LIGHT-loaded AT-MSCs [11].

Finally, local injection of virus-loaded MSCs has also been shown to augment the therapeutic effects of OVs. Babaei et al. examined the potential of AT-MSCs as a novel delivery system for the ReoT3D strain, where IT-administered loaded MSCs were more effective than therapy with ReoT3D and MSCs alone [85]. Local injection of MxV-loaded AT-MSCs was also shown to slow the progression of GL261 murine glioblastoma model. In vitro, loaded AT-MSCs were capable of passing artificial blood–brain barrier (BBB) [86]. Similarly, intracranial injections of AT-MSCs infected with MxV were able to abolish orthotopic U87 tumor growth [87]. AT-MSCs also support VV replication and hypothetically can be used to deliver this oncolytic virus [88].

A large number of studies have demonstrated the chemotaxis of MSCs to injured tissues and tumor sites [89,90,91]. MSCs adhere to TNF-α activated endothelium through VCAM-1 and transmigrate using both leukocyte-similar and unique mechanisms. Interestingly, compared to leukocytes, transmigration occurred at a slower pace, taking several hours [92]. MSC recruitment to tumor sites is facilitated through numerous factors secreted by stroma, including growth and angiogenic factors (PDGF, FGF, VEGF, SDF, HGF), chemokines, and inflammatory cytokines (CCL2, CCL5, CCL22 and CXCL12, TNFα, TGFβ, IL-1β, IL-6, IL-8) [91,93]. Accumulation of oAd and MV-infected MSCs in the liver and lung tumors after IV infusion was reported in several studies, suggesting that infection did not impede the homing capability, though the situation can be different for other OVs [65,67,74,93]. Additionally, culture conditions have significant effects on MSC chemotaxis, offering strategies to improve homing. For instance, the addition of TNF-α to culture media was found to increase expression of chemokine receptors including CCR2, CCR3, and CCR4 [93,94].

The size of MSCs strongly limits migration efficiency upon IV administration. MSCs are relatively large cells compared to monocytes, the largest cells in the bloodstream (diameter 15–30 μm vs. 12–20 μm, respectively), which predisposes them to entrapment in small blood vessels [95,96,97]. As shown in several studies, the vast majority of BM-MSCs accumulated in pulmonary capillaries shortly after IV infusion, being later redistributed to the liver and kidneys [98,99]. Notably, pretreatment with vasodilators before injection improved MSC homing to the long bones and decreased the number of MSCs trapped in lungs, concomitant with faster increase in MSCs numbers in liver [98]. Nevertheless, IV-infused MSCs can be successfully used for targeting lung and liver tumors.

### 3.2. Neural Stem Cells (NSCs)

NSCs are multipotent progenitors of neurons, astrocytes, and oligodendrocytes, and are present in the adult brain in specific areas. The focus on NSC transplantation for therapeutic purposes was set in the field of regenerative medicine. Later, NSCs emerged as OV carriers, primarily for brain malignancies.

Autologous NSCs can be obtained after differentiation of pluripotent stem cells or lineage reprogramming of somatic cells. While both of these approaches are time- and labor-costly, immortalized NSC lines represent an alternative option. Certain safety risks associated with therapeutic application of immortalized lines include potential of secondary malignancies. It was reported that intranasal delivery of HB1.F3-effluc NSC line led to formation of lung tumors in BALB/c nude mice [100]. However, the majority of available NSC lines have not been found to generate tumors in immunodeficient animals [101]. The FDA-approved, immortalized NSC line HB1.F3.CD21 was widely used for several therapeutic approaches in glioma treatment, including gene therapy and OV delivery in both preclinical and clinical settings [102].

NSCs have been extensively studied as carriers for CRAd-S-pk7, a glioma-targeted oAd engineered with a Survivin promoter and a pk7 fiber modification to enhance tumor tropism. The HB1.F3.CD21 cell line supports CRAd-S-pk7 replication and NSC lysis was observed at high virus doses after 2–3 days of culture [103]. Intracranial injection of loaded NSCs in orthotropic glioma models improved retention of virus in the tumor site and increased survival of mice compared to neat virus [103,104,105].

This approach was further translated into a clinical setting. In a cohort of 11 patients with glioblastoma and 1 patient with anaplastic astrocytoma, CRAd-S-pk7-loaded NSCs were injected into the resection cavity following standard chemotherapy. Though no dose-limiting toxicity was reached, one patient developed viral meningitis due to unintentional injection into the lateral ventricle. Progression-free survival (PFS) and overall survival (OS) did not reach confidence intervals but were slightly higher than historical controls [106].

The combination of HB1.F3.CD21 and CRAd-S-pk7 was also evaluated in models of ovarian cancer. Upon IP infusion, infected NSCs slowed the progression of orthotopically implanted OVCAR8 and ID8 tumors and protected the OV from neutralizing antibodies. Treatment with cisplatin and CRAd-S-pk7-loaded NSCs resulted in more pronounced inhibition of tumor growth compared to therapy with cisplatin or NSCs alone [107]. In another study using the same NSC line and administration route, HB1.F3.CD21 was used to deliver the conditionally replicating orthopoxvirus, CF33. Like CRAd-S-pk7, CF33 was able to induce NSC lysis, but after 4–5 h post infection, the majority of NSCs maintained viability and migration capacity. CF33-loaded NSCs were able to improve retention of CF33 in tumors [108]. It was also shown that human NSCs and the HB1.F3.CD21 line support replication of MxV, suggesting that this OV can be “compatible” with NSC-mediated delivery [109].

Both endogenous and cell culture-derived NSCs, including the aforementioned immortalized lines, demonstrate remarkable tumor tropism [110,111]. Upon intracranial implantation in cerebral hemispheres of mice, contralaterally to established U87 xenograft tumors, NSCs were able to cross the midline and migrate to the tumor-bearing hemisphere. Notably, the CRAd-S-pk7 modification enhanced NSC homing to the tumor upon intracranial administration through upregulation of CXCR4 and VEGFR2 on NSCs [104]. NSC chemotaxis toward glioma and CNS tumors is partially regulated by factors that are also involved in neural stem cell migration during development. For example, stem cell factor (SCF) and its receptor c-Kit act as a chemotactic axis for NSCs [104] CXCR4 on NSCs acts as a receptor for CXCL12, chemokine produced by astrocytes and endothelium upon inflammatory conditions [112]. CXCR4 overexpression in CRAd-S-pk7 loaded NSCs has shown promise in non-invasive targeting of brain tumors through intranasal administration route, though irradiation of intracranial tumors to induce CXCL12 release was required to achieve efficient migration [113].

Several lines of evidence support the tumor-directed migration of non-infected NSCs following IV infusion. For example, in a mouse model of disseminated neuroblastoma, Abody et al. demonstrated that NSCs were able to reach micrometastases in the liver and bones [114,115]. Similarly, when NSCs were used as vehicles for therapeutic antibodies, they successfully localized to tumor sites in an orthotopic breast cancer model [116]. In ovarian cancer, IP implantation resulted in more favorable NSC distribution compared to the IV route, as few NSCs were detected in tumors within the peritoneal cavity following IV administration [117]. IV-delivered NSCs are prone to becoming trapped in peripheral tissues [118]. Compared to MSCs, NSCs have shown a twofold greater ability to pass through the lung vessels [119]. Still, limitations related to NSC size should still be taken into account when considering this carrier for IV administration.

### 3.3. Blood Outgrowth Endothelial Cells (BOECs)

BOECs are fully differentiated, proliferative endothelial cells derived from endothelial colony-forming cells (ECFCs). ECFCs can be isolated from a variety of sources, including AT, lung tissue, UCB, and peripheral blood (PB) [120,121,122]. The most accessible source for autologous cell transfer requires long-term culturing of the peripheral blood mononuclear cell (PBMC) fraction, allowing to obtain a pool of BOECs in about 3 weeks [123,124].

BOECs were described as carriers of oncolytic modified VSV strains expressing human/mouse IFN-β. Human BOECs supported VSV-IFN-β replication and reduced tumor burden in an A549 SCID mice model, though overall survival was not improved compared to neat virus [125]. In a metastatic LM2 mammary tumor model, successful targeting of lung tumors by murine BOECs was observed, with carriers persisting in lung tissue 24 h after IV injection [125].

The potential of BOECs as OV carriers remains understudied. The isolation of BOECs from peripheral blood is relatively noninvasive, and the ability to obtain sufficient numbers of carriers can be considered a potential advantage for IV delivery. However, BOECs can contribute to enhanced tumor growth through modulation of tumor angiogenesis at early stages, as reported for both immunocompromised and immunocompetent mice models [126,127]. Therefore, in the context of OV delivery, induction of cell death in the vast majority of BOECs after infection is desirable. Shortly after IV injection, BOECs accumulate in the lungs, liver, and tumor tissue. The incorporation of BOECs to tumor vasculature was observed by 24th day post injection [127]. It is generally accepted that BOECs migrate to sites of neovascularization through the VEGF–VEGFR axis, though concentration-dependent migration of BOECs was also reported for CXCL12 [128,129].

### 3.4. Cancer Cell Lines and Other Immortalized Cell Lines

Cancer cell lines in the role of carriers have two major advantages—they can be easily obtained in large quantities and are often highly susceptible to OV infection. Several human and murine cancer lines of epithelial origin were evaluated as carriers in different settings. IT injection of oAd-infected A549 cells was curative in 90% of oAd-immune mice with syngeneic squamous cell carcinoma cell tumors, with nearly no effect achieved by injections of the virus [48]. Another study successfully utilized MC38 cancer cell line for VV delivery upon IP injection in pre-immunized MC38 peritoneal carcinomatosis model. Notably, infected carriers were not able to form tumors in immunocompetent mice, being lysed within 3 days post-infection [37].

IV administration of VSV-infected A549 and CT26 cell lines in immunocompetent mice models with CT26 lung tumors led to accumulation of carriers in lungs. Though the viral payload was successfully delivered to recipient cancer cells, the similar biodistribution patterns in tumor-free mice suggest that transfer of OV occurred stochastically, rather than due to directed migration of these carriers [29]. A modified Morris hepatoma cell line MH infected with oncolytic H-1 parvovirus decreased the number of MH lung metastases in a rat model [130].

Similarly to other carriers of non-hematological origin, cell size remains one of the main limitations—most of the systemically administered epithelial cells tended to terminally reside within the capillary system of lungs [130]. Leukemia cell lines, however, were able to reach subcutaneous tumors and disseminated lymphoma/myeloma lesions. As shown by Power et al., VSV-infected L1210 cells were able to deliver virus to flank-located subcutaneous tumors upon IV infusion [29]. This route was also used for MV-infected U937 cells and was successful in a disseminated lymphoma model. In IP ovarian and hepatocellular cancer xenografts, locally delivered MV-U937 transferred virus to the lesions in the presence of neutralizing antibodies and prolonged survival of treated mice after repeated injections [131]. Remarkably, preferential homing of myeloma cell lines to the bone marrow through the CXCR4-CXCL12 axis can be utilized for reaching BM-located tumor sites [132]. MV-infected myeloma cell line MM.1 was efficacious in the disseminated myeloma model and retained capacity to replicate MV after sublethal irradiation [133]. Supporting these findings, murine VSV-infected 5TGM1 myeloma cells were able to deliver virus to the sites of myeloma growth [134].

The human mono-mac-6 monocytic cell line and Syrian hamster HM-1 macrophage cell line were both susceptible to oAd-GFP infection and improved virus targeting to the tumor in nude mice, though the majority of carriers was accumulated in the liver. In Syrian hamsters, liver entrapment of HM-1 was even more pronounced, making delivery to the tumor completely inefficient [47].

The NK-92 cell line has been used to deliver coxsackievirus A7 (CVA7) in the subcutaneous LN229 glioma xenograft model, where IV infusion significantly reduced tumor volume. Similarly to MM1, sublethal irradiation of these carriers did not restrict replication of CVA7 [135]. Notably, NK-92 inoculation in nude mice did not induce leukemia. Clinical trials that involved infusion of NK-92 have reported a low incidence of side effects [136].

Although propagation in culture quickly generates sufficient numbers of carriers, the innate genetic instability of cancer cell lines limits standardization of the final cell product and raises substantial safety concerns. Sublethal irradiation of carriers before administration or integration of kill-switch systems are precautionary measures that can be taken to minimize the risk of secondary malignancies, especially in patients that already experience immunosuppressive state. While sublethal irradiation does not affect the replicative activity of some OVs, the versatility of this approach remains unclear [133,135].

## 4. Immune Cells as OV Carriers

### 4.1. T-Cells and Cytokine-Induced Killer (CIK) Cells

In the simplest setting, naive autologous T-cells can be obtained from patients’ blood during the standard procedure of leukapheresis, followed by positive selection. Approaches that utilize antigen-specific T cells, tumor-infiltrating lymphocytes (TILs), and CIKs require the activation and long-term expansion of carriers in GMP-compliant conditions, increasing the cost of therapy.

T-cells are largely non-permissive for OV infection and possess another modality of virus carriage called hitchhiking, being able to transfer surface-bound infectious particles to tumor cells either passively or upon tight contact in immunologic synapses. Several factors were suggested to influence the efficiency of hitchhiking, including activation status of T-cells and loading dose [137,138].

Cross-infection of target cells after contact with NDV-loaded T-cells occurred more efficiently when activated T-cells were used, suggesting that improved transfer was associated with increased frequency of immunological synapses [137]. As shown for retroviral particles, efficient virus hand-off was also associated with increased heparinase expression by antigen-specific CD8+ T-cells. Notably, retroviral hitchhiking strongly improved the efficiency of T-cell immunotherapy by increasing antigen repertoire through direct lysis of cancer cells due to viral infection. In mice bearing tumors without relevant antigen, survival was severely impaired, suggesting that T-cell mediated killing, rather than virus hand-off largely accounted for therapeutic effect. [138]. In another study, IP administration of HSV-loaded antigen-specific lymphocytes increased the survival rate of mice bearing MC26 peritoneal metastases compared to neat virus and plain tumor antigen-specific lymphocytes. Survival was unaffected when non-antigen specific T-cells were used [139].

Despite similar mechanisms that are likely to occur upon viral particle absorption and shedding, in vitro kinetics of the hitchhiking process reported to be different for retroviruses (24–72 h), NDV (6–12 h), and Vaccinia virus (48–72 h) [137,140]. The extent of virus protection from neutralizing antibodies during hitchhiking remains an open question. In a study by Ilett et al., naive T-cells loaded with ReoT3D were able to prime anti-tumor immune responses and reduce metastatic burden in tumor-draining LNs of both naive and reovirus-immune B16 tumor models, suggesting that the activation status of T-cells does not critically affect the hitchhiking efficiency of reovirus. Unexpectedly, only T-cells loaded at 0.1 MOI were able to facilitate transfer in immune animals [141]. This finding was supported by study in VSV-loaded T-cells, setting a threshold of <1 MOI, when higher doses completely abolished delivery and therapeutic effects, providing additional unwanted immunization of mice [142]. In subcutaneous or disseminated human myeloma xenografts, MV-loaded activated T-cells failed to transfer virus in passively immunized mice [44].

Apart from hitchhiking, B7-H3 CAR-T cells were able to replicate HSV without strong impairment of cytotoxic activity. Notably, IV administration of loaded CAR-T cells reduced tumor burden in both orthotopic and subcutaneous glioblastoma models [143].

T-cells display the perfect parameters in terms of size and circulation, being typically 5–10 μm in diameter. Naive T cells constitutively express chemokine receptors, particularly CCR7, which binds to the chemokines CCL19 and CCL21, having the potential to reach metastases in secondary lymphoid organs. ReoT3D-loaded naive T-cells were capable of reaching tumor-draining LNs [141]. VSV-loaded naive T-cells provided purification of LN and spleen-localized B16 metastases in virus-immune mice [42]. Activated T-cells innate homing to the sites of inflammation and tissue injury suggests efficient delivery of OV to the tumor, though the number of T-cells that will eventually reach the tumor is limited. T-cell exclusion from tumor microenvironment due to physical tumor barriers and immunosuppressive signaling represents a major hurdle for T-cell mediated OV delivery [144,145].

As shown for activated, MV-loaded T-cells six hours after cell infusion, 40% of T cells were found in the liver, 12% in lungs, 2% in the spleen, and 1.5% in the tumor [44]. For ovalbumin (OVA)-specific CD8 T-cells, 5–14% of adoptively transferred T cells were able to reach B16-OVA tumors [142]. Since this study utilizes a highly immunogenic B16-OVA tumor model, trafficking rates are likely to be lower in real-world settings.

Cytokine-induced killer (CIK) cells are a heterogeneous population of immune effector cells with a mixed T-cell and NK-cell phenotype, exhibiting MHC-unrestricted cytotoxicity. These cells are generated through ex vivo incubation of PBMCs with interferon-gamma, CD3-specific antibody, and interleukin-2 [146]. In contrast to T-cells, CIK cells were able to support productive replication of MV without impairment of cytotoxic activity, and prolonged survival in mice with disseminated KAS-6/1 myeloma after IV administration [147]. Synergetic effects of IV-infused, VV-infected CIK cells were shown in IP ovarian tumor xenografts of nude mice and in immunocompetent mice bearing orthotopic breast cancer JC tumors. VV replication kinetics followed unusual patterns, with an extended eclipse period up to 48 h [140]. The ability of CIK cells to internalize these OVs offers superior virus protection compared to T-cell hitchhiking [140].

### 4.2. Monocytes and Macrophages

Monocytes and macrophages often serve as “essential” carriers that facilitate virus dissemination throughout the body during naturally occurring viral infections. A broad range of viruses, including members of Paramyxo-, Orthomyxo-, Flavi-, Picorna-, Rhabdoviridae families, can initiate productive or latent infection in monocytes [148].

Autologous monocytes can be rapidly recovered in large numbers from PB after leukapheresis and are amenable to differentiation in vitro into macrophages. Monocytes are permissive to infection with oncolytic HSV-1 carrying deletions similar to T-VEC, though replication activity was minimal. Loaded monocytes were able to transfer viral payload in vitro upon co-culture and accumulated in chorioallantoic membrane-implanted UM-SCC-11B tumors [149].

The encapsulation of oAd into CCL2-coated liposomes that were preferentially taken up by CCR2-expressing monocytes was able to significantly reduce tumor size and pulmonary metastasis burden in prostate cancer-bearing mice [150]. Monocytes were also suggested as potential carriers for MV, being productively infected, though the percentage of MV-positive cells was lower compared to monocyte-derived dendritic cells (moDC) in the same study [151]. An interesting mechanism of carriage was described for ReoT3D, where monocytes were able to internalize reovirus-antibody complexes through Fc-receptors and further deliver infectious particles to subcutaneous B16 tumors, despite complete initial neutralization [45].

Several studies also utilized macrophages as carriers. In particular, human monocyte-derived macrophages supported oAd replication and inhibited regrowth and metastatic spread of ectopic LnCaP tumors in nude mice after docetaxel treatment or radiation [152]. In the immunocompetent model, HSV-infected BM-derived macrophages displayed similar effectiveness in prolonging survival compared to free virus in orthotopic mammary cancer models and stereotactically implanted brain metastases [153]. Both studies utilized the IV route of carrier administration.

The IV-administered monocytes and macrophages share similar biodistribution patterns, being initially accumulated in liver, spleen, and lungs with later partial relocation to tumors [154]. Recruitment of myeloid cells to the tumor site is a well-documented phenomenon, with significant implications for immune evasion. While being detrimental in a natural setting, this signaling may serve as bait for infected cell carriers, given that virus-loaded monocytes retain migratory capacity. An array of tumor-secreted chemokines facilitates migration of monocytes, as well as survival and differentiation into tumor-associated macrophages [155]. Major receptor/ligand axes supporting monocyte trafficking include M-CSF/CSF-1R, CCL2/CCR2, CX3CL1/CX3CR1, CCL5/CCR5. Of these, CCL2/CCR2 is of particular importance, as shown in mouse xenograft models for CCR2+ classical human monocytes [156,157,158,159,160].

### 4.3. Myeloid-Derived Suppressor Cells (MDSCs)

Myeloid-derived suppressor cells (MDSCs) represent a rare, heterogeneous population of myeloid origin with potent immunosuppressive properties. Both murine and human MDSCs can be divided into two functionally and phenotypically distinct subsets: polymorphonuclear and monocytic MDSCs [161]. In humans, the expression of monocytic CD14 or granulocytic CD15, along with low HLA-DR, helps to distinguish these MDSC subsets [162,163]. More specific biomarkers for MDSCs are still not described, though LOX-1 and S100A9 were suggested as additional auxiliary markers [157]. MDSCs as a part of circulating PBMCs are barely detected in healthy individuals. In cancer patients, MDSCs expand due to alteration of myelopoesis under chronic inflammation facilitated by tumor-secreted chemokines, especially at late disease stages [155,158]. Furthermore, normal myeloid cells can be converted into MDSCs by extracellular vesicles released from cancer cells [164,165,166,167].

MDSCs were investigated as carriers for systemic administration of modified VSV strain in a metastatic colon cancer model, prolonging long-term survival compared to free VSV [167]. As a key component of the tumor immunosuppressive environment, MDSCs are actively recruited to the tumor site. Similarly to monocytes, MDSC recruitment largely depends on CCR2/CCL2 signaling, while additional chemokines such as CCL3 and CCL5 also contribute to MDSC migration and expansion in BM [168,169,170]. MDSC tumor tropism may be superior to CIKs, activated T-cells, naive T-cells, monocytes, macrophages, and dendritic cells [167].

Major limitations of MDSCs as potential carriers include their rarity, short lifespan and sensitivity to cryopreservation [170]. In addition, characterization of MDSCs is complicated due to a lack of specific cell-surface markers and requires functional assays. Frequency of MDSCs in PB is affected by isolation techniques and shows significant variation across different cancer types. In patients with breast cancer, head and neck cancer and colorectal cancer, the percentage of polymorphonuclear MDSCs ranged from 0.5 to 10%. In contrast, the increase in polymorphonuclear MDSCs was not detected in patients with melanoma, compared to healthy donors [171]. Generation of MDSCs in vitro from bone marrow precursors was reported through culture with GM-CSF and IL-6 [172]. Another example included the generation of monocytic MDSC-like cells with immunosuppressive functions after culturing peripheral blood monocytes with GM-CSF, IL-4, and PGE2 [173]. Further development of such approaches may support MDSC application in the role of cell carriers.

### 4.4. Dendritic Cells (DCs)

Dendritic cells represent a heterogeneous population of professional antigen-presenting cells that act as a bridge between the innate and adaptive immune responses. Most of the current knowledge about human DC biology has been obtained through in vitro models of differentiation from hematopoietic stem cells isolated from BM and subsequently stimulated with GM-CSF, or monocytes stimulated with GM-CSF and IL-4. PBMCs represent the most accessible source for DC generation in vitro for both research and therapeutic purposes (e.g., in the development of dendritic vaccines). The production of DC from monocytes requires cultivation for 5–6 days in the presence of IL-4 and GM-CSF to form immature moDCs. Shorter protocols allow generation of functional moDCs pool in 3 days [174].

DCs have emerged as OV carriers in several studies. Murine BMDCs supported replication of ReoT3D. IV injection of ReoT3D-loaded BMDCs successfully purged metastatic sites in LNs of reovirus-immune mice. Notably, immature BMDCs were less efficient in terms of trafficking to LNs, despite CCR7 expression. In addition, reovirus loading even at high MOI did not induce cell death of all BMDCs, with the latter being capable to prime anti-tumor immune response [43]. In parallel, human moDCs also internalized ReoT3D and were capable of transferring infectious virus particles to target culture in the presence of neutralizing serum [141]. Ilett et al. used immature human moDCs to deliver MV in an immunocompromised murine model of human KAS 6/1 myeloma. Human moDCs were highly susceptible to MV-eGFP infection, irrespective of their maturation status, with the percentage of infected cells ranging from 40 to 80%. This treatment extended survival, but was not curative [151]. In another study, both IV and intrapleural MV-infected moDC injections were able to prevent accumulation of the pleural exudate in malignant pleural effusion xenograft model of advanced breast cancer [175]. In the subcutaneous prostate cancer model, IV-infused BMDCs infected with recombinant oAds Ad-PPC-NCS and Ad-PPC-rmhTNF significantly inhibited tumor growth and increased survival time [176].

Due to technical availability and large yields, mouse BMDCs are commonly used as carriers in immunocompetent models, while human moDCs were validated in vivo as carriers in immunocompromised murine models. It is important to note that human moDCs and murine BMDCs represent distinct, functionally non-equivalent subpopulations. As reported, mouse BMDCs themselves comprise a phenotypically heterogeneous mixture of conventional DCs and macrophage-like cells [177]. The resulting population is affected by culture conditions—while common protocol suggests cultivation of BM cells with GM-CSF, which favors the expansion of common monocyte precursors, expansion of common dendritic cell precursors was also observed, leading to ontogenetically distinct cells in the resulting progeny. Importantly, the addition of IL-4 limits the generation of macrophages. Several culture protocols suggested the addition of Flt3L, which supports generation of conventional DCs with cDC2 phenotype [178,179]. There is a possibility that therapeutic effects observed upon using BMDCs as carriers, are dependent on the T-cell priming by these cells, apart from the improved OV delivery [176].

Key differences between DC subsets and activation states in the context of OV delivery lie in their distinct migration patterns and homing ability to lymphoid tissues. While conventional immature DCs are non-migratory and reside in peripheral tissues before encountering pathogens [180,181], human immature moDCs express CCR2, CCR5 and selectively migrate to inflammatory sites in a monocyte-like manner [182]. Mature DCs upregulate CCR7 and can be considered for LN-selective delivery. Murine BMDCs display pronounced homing ability to LNs [183,184] while mixed findings were reported for human moDCs. CCR7 expression was detected on human moDCs studied in vitro [185,186,187]. In contrast, murine lung-resident moDCs have increased repressive histone modifications at the Ccr7 locus and fail to upregulate CCR7 after LPS-induced maturation [188]. Of note, moDCs detected in lymphoid organs lack CCR7 expression, suggesting alternative recruitment pathways [189,190,191]. In acute inflammation, mo-DCs in LNs originate from monocytes that can be directly recruited from blood rather than from peripheral tissues [191].

## 5. Comparative Analysis and Selection of Cell Carriers

**Figure 2:**
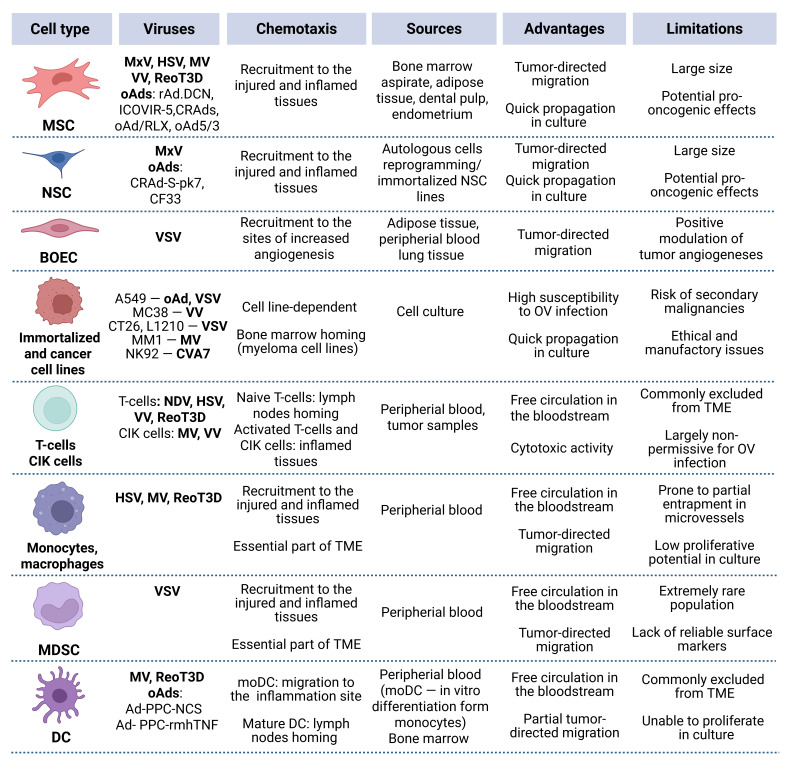
Overview of the key characteristics of various cell carrier types. Main advantages and limitations are outlined with respect to IV administration.

The concept of using cell carriers for OV delivery led to an array of promising proof-of-concept studies. Based on the aforementioned studies, diverse cell types were successful in different in vivo cancer models (Figure 2).

**Figure 3:**
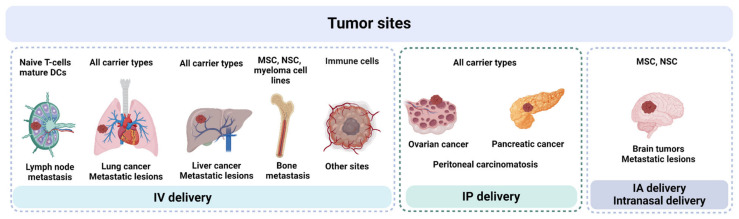
Recommendations for selecting administration routes and cell carrier types based on preclinical models, chemotactic properties, and ability to traverse microvasculature.

For some widely investigated OVs (e.g., VV), several carrier types have been proposed, raising the inevitable question: which cell type can be considered the most promising? A few studies have directly compared carriers, highlighting features that may be useful in specific models—for example, superior trafficking of reovirus-loaded mature DCs to affected LNs [41]. On the other hand, mature DCs generally lack tumor-specific chemotaxis, suggesting that other carrier types may be more effective in reaching solid tumors. Therefore, the localization of the primary tumor and/or metastatic lesions is one of the most important factors to consider upon selection of both cell carrier type and administration route (Figure 3).

Upon IV administration, any type of carrier cells is likely to reach lesions in the lungs or liver. Mechanical entrapment of large-sized carriers in these locations can be viewed as a potential way to retain large yields of infectious OV particles in close proximity to tumor sites. Similarly, IP injection of carrier cells is applicable for combating ovarian cancer, pancreatic cancer and peritoneal carcinomatosis, as shown in numerous studies [11,82,83,107].

Immune cells represent a heterogeneous subclass of cell carriers characterized by remarkable migratory capacity and free circulation in the bloodstream. Direct therapeutic effects of cell transfer therapies led to the development of combined approaches, utilizing immune cells in the role of both carriers and inducers of anti-tumor immune response. Immune cells can be roughly considered as “all-purpose” cell carriers with ability to reach distant tumor sites upon IV infusion [140,167,176]. Still, further studies are needed to fully evaluate the potential of these carriers. In cases of metastatic spread to lymph nodes, T-cells and mature DCs stand out as promising carriers due to their LN-homing ability [42,43,141]. Although technically more demanding, intranodal administration of carriers may provide a dual-pronged attack on cancer cells, boosting both antitumor immunity and T-cell priming in non-affected lymph nodes. MSCs, NSCs, and myeloma cell lines can be considered for targeting bone metastases, which remain the most challenging location for most of the anticancer therapies [98]. For intracranial tumors, IA and IT administration of NSC and MSC carriers seems to be the most appropriate setting, though successful attempts to reach tumors using intranasal delivery of NSCs were also reported [192,193].

## 6. Conclusions

The versatility of cell-based delivery options allows tailored approaches, with the choice of carrier and administration route adapted to the specific tumor setting. It is important to note that real-world application of cell carriers will require ex vivo expansion of cells under GMP-compliant environments, leading to high costs along with logistical and regulatory challenges. Nevertheless, successful implementation of CAR-T cell therapies has paved the way for fast-paced development of cell-based therapeutic products. Landmark clinical trials featuring oAd-loaded MSCs and NSCs bring hope for cell-based OV delivery translation from bench to bedside despite the obvious current restrictions associated with cell therapies [50,75,106]. Supporting this, an ongoing clinical trial features AT-MSCs as carriers of oncolytic modified MV through IP route in patients with recurrent ovarian, primary peritoneal, or fallopian tube cancer (NCT02068794). In another currently recruiting study, allogeneic BM-MSCs loaded with the oncolytic adenovirus DNX-2401 are administered by IA injection in patients with recurrent glioblastoma, IDH-mutant astrocytoma, gliosarcoma, or wild-type IDH anaplastic astrocytoma (NCT03896568).

Given the urgent need for improving systemic delivery of OVs, cell carriers harness the potential to create game-changing shift in the field of virotherapy. Still, there is always room for improvement, such as approaches to increase OV uptake by carriers, the augmentation of tumor-specific chemotaxis, and the expansion of potential carrier spectra, all of which hold promise for the development of more advanced therapies. Detailed investigation of triple tumor-carrier-virus crosstalk will highlight new strategies for more efficient cell carrier applications that combine targeted OV delivery and amplification of anticancer immune response.

## References

[1] Lin D., Shen Y., Liang T. (2023). Oncolytic virotherapy: Basic principles, recent advances and future directions. Signal Transduct. Target. Ther..

[2] Norman K.L., Lee P.W. (2000). Reovirus as a novel oncolytic agent. J. Clin. Investig..

[3] Nemunaitis J. (2002). Live viruses in cancer treatment. Oncology.

[4] Heise C., Kirn D.H. (2000). Replication-selective adenoviruses as oncolytic agents. J. Clin. Investig..

[5] Msaouel P., Iankov I.D., Dispenzieri A., Galanis E. (2012). Attenuated oncolytic measles virus strains as cancer therapeutics. Curr. Pharm. Biotechnol..

[6] Kirn D. (2000). Replication-selective oncolytic adenoviruses: Virotherapy aimed at genetic targets in cancer. Oncogene.

[7] Takano G., Esaki S., Goshima F., Enomoto A., Hatano Y., Ozaki H., Watanabe T., Sato Y., Kawakita D., Murakami S. (2021). Oncolytic activity of naturally attenuated herpes-simplex virus HF10 against an immunocompetent model of oral carcinoma. Mol. Ther. Oncolytics.

[8] Hosseini M., Farassati F.S., Farassati F. (2020). Targeting Cancer Stem Cells by Oncolytic Viruses and Nano-Mediated Delivery. OncoTargets Ther..

[9] Eriksson M., Guse K., Bauerschmitz G., Virkkunen P., Tarkkanen M., Tanner M., Hakkarainen T., Kanerva A., Desmond R.A., Pesonen S. (2007). Oncolytic adenoviruses kill breast cancer initiating CD44+CD24-/low cells. Mol. Ther..

[10] Chaurasiya S., Chen N.G., Warner S.G. (2018). Oncolytic Virotherapy versus Cancer Stem Cells: A Review of Approaches and Mechanisms. Cancers.

[11] Jazowiecka-Rakus J., Sochanik A., Hadryś A., Fidyk W., Chmielik E., Rahman M.M., McFadden G. (2022). Combination of LIGHT (TNFSF14)-Armed Myxoma Virus Pre-Loaded into ADSCs and Gemcitabine in the Treatment of Experimental Orthotopic Murine Pancreatic Adenocarcinoma. Cancers.

[12] Hwang J.K., Hong J., Yun C.O. (2020). Oncolytic Viruses and Immune Checkpoint Inhibitors: Preclinical Developments to Clinical Trials. Int. J. Mol. Sci..

[13] Wang Y., Zhu M., Chi H., Liu Y., Yu G. (2024). The combination therapy of oncolytic virotherapy. Front. Pharmacol..

[14] El-Sayes N., Walsh S., Vito A., Reihani A., Ask K., Wan Y., Mossman K. (2022). IFNAR blockade synergizes with oncolytic VSV to prevent virus-mediated PD-L1 expression and promote antitumor T cell activity. Mol. Ther. Oncolytics.

[15] Liu Z., Ravindranathan R., Kalinski P., Guo Z.S., Bartlett D.L. (2017). Rational combination of oncolytic vaccinia virus and PD-L1 blockade works synergistically to enhance therapeutic efficacy. Nat. Commun..

[16] Chaurasiya S., Fong Y., Warner S.G. (2021). Oncolytic Virotherapy for Cancer: Clinical Experience. Biomedicines.

[17] Bradbury P.A., Morris D.G., Nicholas G., Tu D., Tehfe M., Goffin J.R., Shepherd F.A., Gregg R.W., Rothenstein J., Lee C. (2018). Canadian Cancer Trials Group (CCTG) IND211: A randomized trial of pelareorep (Reolysin) in patients with previously treated advanced or metastatic non-small cell lung cancer receiving standard salvage therapy. Lung Cancer.

[18] Cohn D.E., Sill M.W., Walker J.L., O’Malley D., Nagel C.I., Rutledge T.L., Bradley W., Richardson D.L., Moxley K.M., Aghajanian C. (2017). Randomized phase IIB evaluation of weekly paclitaxel versus weekly paclitaxel with oncolytic reovirus (Reolysin(R)) in recurrent ovarian, tubal, or peritoneal cancer: An NRG Oncology/Gynecologic Oncology Group study. Gynecol. Oncol..

[19] Monga V., Miller B.J., Tanas M., Boukhar S., Allen B., Anderson C., Stephens L., Hartwig S., Varga S., Houtman J. (2021). Intratumoral talimogene laherparepvec injection with concurrent preoperative radiation in patients with locally advanced soft-tissue sarcoma of the trunk and extremities: Phase IB/II trial. J. Immunother. Cancer.

[20] Shalhout S.Z., Miller D.M., Emerick K.S., Kaufman H.L. (2023). Therapy with oncolytic viruses: Progress and challenges. Nat. Rev. Clin. Oncol..

[21] Yoon A.R., Rivera-Cruz C., Gimble J.M., Yun C.O., Figueiredo M.L. (2022). Immunotherapy by mesenchymal stromal cell delivery of oncolytic viruses for treating metastatic tumors. Mol. Ther. Oncolytics.

[22] Raja J., Ludwig J.M., Gettinger S.N., Schalper K.A., Kim H.S. (2018). Oncolytic virus immunotherapy: Future prospects for oncology. J. Immunother. Cancer.

[23] Robert C., Milhem M.M., Sacco J.J., Michels J., In G.K., Muñoz-Couselo E., Schadendorf D., Beasley G.M., Niu J.J., Chmielowski B. (2024). LBA46 Primary efficacy, safety, and survival data from the registration-intended cohort of patients with anti–PD-1–failed melanoma from the IGNYTE clinical trial with RP1 combined with nivolumab. Ann. Oncol..

[24] Tyson M.D., Uchio E.M., Nam J.-K., Joshi S.S., Bivalacqua T.J., Steinberg G.D., Kitamura H., Tran B., Li R. (2025). Final results: Bond-003 cohort c- phase 3, single-arm study of intravesical cretostimogene grenadenorepvec for high-risk bcg-unresponsive non-muscle invasive bladder cancer with carcinoma in situ. J. Urol..

[25] Willmon C., Harrington K., Kottke T., Prestwich R., Melcher A., Vile R. (2009). Cell carriers for oncolytic viruses: Fed Ex for cancer therapy. Mol. Ther..

[26] Masurier C., Salomon B., Guettari N., Pioche C., Lachapelle F., Guigon M., Klatzmann D. (1998). Dendritic cells route human immunodeficiency virus to lymph nodes after vaginal or intravenous administration to mice. J. Virol..

[27] Engering A., Van Vliet S.J., Geijtenbeek T.B., Van Kooyk Y. (2002). Subset of DC-SIGN(+) dendritic cells in human blood transmits HIV-1 to T lymphocytes. Blood.

[28] Groeneveldt C., Kinderman P., Griffioen L., Rensing O., Labrie C., van den Wollenberg D.J.M., Hoeben R.C., Coffey M., Loghmani H., Verdegaal E.M.E. (2024). Neutralizing Antibodies Impair the Oncolytic Efficacy of Reovirus but Permit Effective Combination with T cell-Based Immunotherapies. Cancer Immunol. Res..

[29] Power A.T., Wang J., Falls T.J., Paterson J.M., Parato K.A., Lichty B.D., Stojdl D.F., Forsyth P.A., Atkins H., Bell J.C. (2007). Carrier cell-based delivery of an oncolytic virus circumvents antiviral immunity. Mol. Ther..

[30] Groeneveldt C., van den Ende J., van Montfoort N. (2023). Preexisting immunity: Barrier or bridge to effective oncolytic virus therapy?. Cytokine Growth Factor. Rev..

[31] Hill C., Carlisle R. (2019). Achieving systemic delivery of oncolytic viruses. Expert. Opin. Drug Deliv..

[32] Jain R.K., Stylianopoulos T. (2010). Delivering nanomedicine to solid tumors. Nat. Rev. Clin. Oncol..

[33] Prestwich R.J., Errington F., Diaz R.M., Pandha H.S., Harrington K.J., Melcher A.A., Vile R.G. (2009). The case of oncolytic viruses versus the immune system: Waiting on the judgment of Solomon. Hum. Gene Ther..

[34] Fulci G., Breymann L., Gianni D., Kurozomi K., Rhee S.S., Yu J., Kaur B., Louis D.N., Weissleder R., Caligiuri M.A. (2006). Cyclophosphamide enhances glioma virotherapy by inhibiting innate immune responses. Proc. Natl. Acad. Sci. USA.

[35] Lun X.Q., Jang J.H., Tang N., Deng H., Head R., Bell J.C., Stojdl D.F., Nutt C.L., Senger D.L., Forsyth P.A. (2009). Efficacy of systemically administered oncolytic vaccinia virotherapy for malignant gliomas is enhanced by combination therapy with rapamycin or cyclophosphamide. Clin. Cancer Res..

[36] Kambara H., Saeki Y., Chiocca E.A. (2005). Cyclophosphamide allows for in vivo dose reduction of a potent oncolytic virus. Cancer Res..

[37] Guo Z.S., Parimi V., O’Malley M.E., Thirunavukarasu P., Sathaiah M., Austin F., Bartlett D.L. (2010). The combination of immunosuppression and carrier cells significantly enhances the efficacy of oncolytic poxvirus in the pre-immunized host. Gene Ther..

[38] Davola M.E., Mossman K.L. (2019). Oncolytic viruses: How “lytic” must they be for therapeutic efficacy?. Oncoimmunology.

[39] Ricca J.M., Oseledchyk A., Walther T., Liu C., Mangarin L., Merghoub T., Wolchok J.D., Zamarin D. (2018). Pre-existing Immunity to Oncolytic Virus Potentiates Its Immunotherapeutic Efficacy. Mol. Ther..

[40] Cyrelle Ornella M.S., Kim J.J., Cho E., Cho M., Hwang T.H. (2024). Dose Considerations for Vaccinia Oncolytic Virus Based on Retrospective Reanalysis of Early and Late Clinical Trials. Vaccines.

[41] Chen L., Ma Z., Xu C., Xie Y., Ouyang D., Song S., Zhao X., Liu F. (2023). Progress in oncolytic viruses modified with nanomaterials for intravenous application. Cancer Biol. Med..

[42] Qiao J., Kottke T., Willmon C., Galivo F., Wongthida P., Diaz R.M., Thompson J., Ryno P., Barber G.N., Chester J. (2008). Purging metastases in lymphoid organs using a combination of antigen-nonspecific adoptive T cell therapy, oncolytic virotherapy and immunotherapy. Nat. Med..

[43] Ilett E.J., Prestwich R.J., Kottke T., Errington F., Thompson J.M., Harrington K.J., Pandha H.S., Coffey M., Selby P.J., Vile R.G. (2009). Dendritic cells and T cells deliver oncolytic reovirus for tumour killing despite pre-existing anti-viral immunity. Gene Ther..

[44] Ong H.T., Hasegawa K., Dietz A.B., Russell S.J., Peng K.W. (2007). Evaluation of T cells as carriers for systemic measles virotherapy in the presence of antiviral antibodies. Gene Ther..

[45] Berkeley R.A., Steele L.P., Mulder A.A., van den Wollenberg D.J.M., Kottke T.J., Thompson J., Coffey M., Hoeben R.C., Vile R.G., Melcher A. (2018). Antibody-Neutralized Reovirus Is Effective in Oncolytic Virotherapy. Cancer Immunol. Res..

[46] Reagan M.R., Kaplan D.L. (2011). Concise review: Mesenchymal stem cell tumor-homing: Detection methods in disease model systems. Stem Cells.

[47] Bunuales M., Garcia-Aragoncillo E., Casado R., Quetglas J.I., Hervas-Stubbs S., Bortolanza S., Benavides-Vallve C., Ortiz-de-Solorzano C., Prieto J., Hernandez-Alcoceba R. (2012). Evaluation of monocytes as carriers for armed oncolytic adenoviruses in murine and Syrian hamster models of cancer. Hum. Gene Ther..

[48] Hamada K., Zhang T., Desaki J., Nakashiro K., Itoh H., Tani K., Koyama Y., Hamakawa H. (2010). Carrier cell-mediated cell lysis of squamous cell carcinoma cells by squamous cell carcinoma antigen 1 promoter-driven oncolytic adenovirus. J. Gene Med..

[49] Ghasemi Darestani N., Gilmanova A.I., Al-Gazally M.E., Zekiy A.O., Ansari M.J., Zabibah R.S., Jawad M.A., Al-Shalah S.A.J., Rizaev J.A., Alnassar Y.S. (2023). Mesenchymal stem cell-released oncolytic virus: An innovative strategy for cancer treatment. Cell Commun. Signal.

[50] Ruano D., López-Martín J.A., Moreno L., Lassaletta Á., Bautista F., Andión M., Hernández C., González-Murillo Á., Melen G., Alemany R. (2020). First-in-Human, First-in-Child Trial of Autologous MSCs Carrying the Oncolytic Virus Icovir-5 in Patients with Advanced Tumors. Mol. Ther..

[51] Berebichez-Fridman R., Montero-Olvera P.R. (2018). Sources and Clinical Applications of Mesenchymal Stem Cells: State-of-the-art review. Sultan Qaboos Univ. Med. J..

[52] Stanko P., Kaiserova K., Altanerova V., Altaner C. (2014). Comparison of human mesenchymal stem cells derived from dental pulp, bone marrow, adipose tissue, and umbilical cord tissue by gene expression. Biomed. Pap. Med. Fac. Univ. Palacky. Olomouc Czech Repub..

[53] Wang X., Zhao X., He Z. (2021). Mesenchymal stem cell carriers enhance anti-tumor efficacy of oncolytic virotherapy. Oncol. Lett..

[54] Mader E.K., Butler G., Dowdy S.C., Mariani A., Knutson K.L., Federspiel M.J., Russell S.J., Galanis E., Dietz A.B., Peng K.W. (2013). Optimizing patient derived mesenchymal stem cells as virus carriers for a phase I clinical trial in ovarian cancer. J. Transl. Med..

[55] Wang Y., Huso D.L., Harrington J., Kellner J., Jeong D.K., Turney J., McNiece I.K. (2005). Outgrowth of a transformed cell population derived from normal human BM mesenchymal stem cell culture. Cytotherapy.

[56] Borgonovo T., Solarewicz M.M., Vaz I.M., Daga D., Rebelatto C.L., Senegaglia A.C., Ribeiro E., Cavalli I.J., Brofman P.S. (2015). Emergence of clonal chromosomal alterations during the mesenchymal stromal cell cultivation. Mol. Cytogenet..

[57] Wang Y., Yi H., Song Y. (2021). The safety of MSC therapy over the past 15 years: A meta-analysis. Stem Cell Res. Ther..

[58] Afkhami H., Mahmoudvand G., Fakouri A., Shadab A., Mahjoor M., Komeili Movahhed T. (2023). New insights in application of mesenchymal stem cells therapy in tumor microenvironment: Pros and cons. Front. Cell Dev. Biol..

[59] Lim J.-Y., Kim B.-S., Ryu D.-B., Kim T.W., Park G., Min C.-K. (2021). The therapeutic efficacy of mesenchymal stromal cells on experimental colitis was improved by the IFN-γ and poly(I:C) priming through promoting the expression of indoleamine 2,3-dioxygenase. Stem Cell Res. Ther..

[60] Hass R. (2020). Role of MSC in the tumor microenvironment. Cancers.

[61] Davies L.C., Heldring N., Kadri N., Le Blanc K. (2017). Mesenchymal Stromal Cell Secretion of Programmed Death-1 Ligands Regulates T Cell Mediated Immunosuppression. Stem Cells.

[62] Németh K., Leelahavanichkul A., Yuen P.S.T., Mayer B., Parmelee A., Doi K., Robey P.G., Leelahavanichkul K., Koller B.H., Brown J.M. (2009). Bone marrow stromal cells attenuate sepsis via prostaglandin E(2)-dependent reprogramming of host macrophages to increase their interleukin-10 production. Nat. Med..

[63] Rawat S., Gupta S., Mohanty S., Tyagi R.K., Bisen P.S. (2019). Mesenchymal stem cells modulate the immune system in developing therapeutic interventions. Immune Response Activation and Immunomodulation.

[64] Blanc K.L., Dazzi F., English K., Farge D., Galipeau J., Horwitz E.M., Kadri N., Krampera M., Lalu M.M., Nolta J. (2025). ISCT MSC committee statement on the US FDA approval of allogenic bone-marrow mesenchymal stromal cells. Cytotherapy.

[65] Yoon A.R., Hong J., Li Y., Shin H.C., Lee H., Kim H.S., Yun C.O. (2019). Mesenchymal Stem Cell-Mediated Delivery of an Oncolytic Adenovirus Enhances Antitumor Efficacy in Hepatocellular Carcinoma. Cancer Res..

[66] Zhang Y., Liu C., Wang T., Kong F., Zhang H., Yi J., Dong X., Duan H., Tao N., Yang Y. (2022). Therapeutic effects of mesenchymal stem cells loaded with oncolytic adenovirus carrying decorin on a breast cancer lung metastatic mouse model. Mol. Ther. Oncolytics.

[67] Hoyos V., Del Bufalo F., Yagyu S., Ando M., Dotti G., Suzuki M., Bouchier-Hayes L., Alemany R., Brenner M.K. (2015). Mesenchymal Stromal Cells for Linked Delivery of Oncolytic and Apoptotic Adenoviruses to Non-small-cell Lung Cancers. Mol. Ther..

[68] Stoff-Khalili M.A., Rivera A.A., Mathis J.M., Banerjee N.S., Moon A.S., Hess A., Rocconi R.P., Numnum T.M., Everts M., Chow L.T. (2007). Mesenchymal stem cells as a vehicle for targeted delivery of CRAds to lung metastases of breast carcinoma. Breast Cancer Res. Treat..

[69] Jazowiecka-Rakus J., Sochanik A., Rusin A., Hadryś A., Fidyk W., Villa N., Rahman M.M., Chmielik E., Franco L.S., McFadden G. (2020). Myxoma Virus-Loaded Mesenchymal Stem Cells in Experimental Oncolytic Therapy of Murine Pulmonary Melanoma. Mol. Ther. Oncolytics.

[70] Leoni V., Gatta V., Palladini A., Nicoletti G., Ranieri D., Dall’Ora M., Grosso V., Rossi M., Alviano F., Bonsi L. (2015). Systemic delivery of HER2-retargeted oncolytic-HSV by mesenchymal stromal cells protects from lung and brain metastases. Oncotarget.

[71] Pereboeva L., Komarova S., Mikheeva G., Krasnykh V., Curiel D.T. (2003). Approaches to utilize mesenchymal progenitor cells as cellular vehicles. Stem Cells.

[72] Conget P.A., Minguell J.J. (2000). Adenoviral-mediated gene transfer into ex vivo expanded human bone marrow mesenchymal progenitor cells. Exp. Hematol..

[73] Na Y., Nam J.P., Hong J., Oh E., Shin H.C., Kim H.S., Kim S.W., Yun C.O. (2019). Systemic administration of human mesenchymal stromal cells infected with polymer-coated oncolytic adenovirus induces efficient pancreatic tumor homing and infiltration. J. Control. Release Off. J. Control. Release Soc..

[74] Ong H.T., Federspiel M.J., Guo C.M., Ooi L.L., Russell S.J., Peng K.W., Hui K.M. (2013). Systemically delivered measles virus-infected mesenchymal stem cells can evade host immunity to inhibit liver cancer growth. J. Hepatol..

[75] Garcia-Castro J., Alemany R., Cascallo M., Martinez-Quintanilla J., Arriero Mdel M., Lassaletta A., Madero L., Ramirez M. (2010). Treatment of metastatic neuroblastoma with systemic oncolytic virotherapy delivered by autologous mesenchymal stem cells: An exploratory study. Cancer Gene Ther..

[76] Yong R.L., Shinojima N., Fueyo J., Gumin J., Vecil G.G., Marini F.C., Bogler O., Andreeff M., Lang F.F. (2009). Human bone marrow-derived mesenchymal stem cells for intravascular delivery of oncolytic adenovirus Delta24-RGD to human gliomas. Cancer Res..

[77] Cui L.L., Kerkelä E., Bakreen A., Nitzsche F., Andrzejewska A., Nowakowski A., Janowski M., Walczak P., Boltze J., Lukomska B. (2015). The cerebral embolism evoked by intra-arterial delivery of allogeneic bone marrow mesenchymal stem cells in rats is related to cell dose and infusion velocity. Stem Cell Res. Ther..

[78] Sanchez-Diaz M., Quiñones-Vico M.I., Sanabria de la Torre R., Montero-Vílchez T., Sierra-Sánchez A., Molina-Leyva A., Arias-Santiago S. (2021). Biodistribution of Mesenchymal Stromal Cells after Administration in Animal Models and Humans: A Systematic Review. J. Clin. Med..

[79] Na Kim H., Yeol Kim D., Hee Oh S., Sook Kim H., Suk Kim K., Hyu Lee P. (2017). Feasibility and Efficacy of Intra-Arterial Administration of Mesenchymal Stem Cells in an Animal Model of Double Toxin-Induced Multiple System Atrophy. Stem Cells Transl. Med..

[80] Du W., Seah I., Bougazzoul O., Choi G., Meeth K., Bosenberg M.W., Wakimoto H., Fisher D., Shah K. (2017). Stem cell-released oncolytic herpes simplex virus has therapeutic efficacy in brain metastatic melanomas. Proc. Natl. Acad. Sci. USA.

[81] Duebgen M., Martinez-Quintanilla J., Tamura K., Hingtgen S., Redjal N., Wakimoto H., Shah K. (2014). Stem cells loaded with multimechanistic oncolytic herpes simplex virus variants for brain tumor therapy. J. Natl. Cancer Inst..

[82] Komarova S., Kawakami Y., Stoff-Khalili M.A., Curiel D.T., Pereboeva L. (2006). Mesenchymal progenitor cells as cellular vehicles for delivery of oncolytic adenoviruses. Mol. Cancer Ther..

[83] Mader E.K., Maeyama Y., Lin Y., Butler G.W., Russell H.M., Galanis E., Russell S.J., Dietz A.B., Peng K.W. (2009). Mesenchymal stem cell carriers protect oncolytic measles viruses from antibody neutralization in an orthotopic ovarian cancer therapy model. Clin. Cancer Res..

[84] Jazowiecka-Rakus J., Hadrys A., Rahman M.M., McFadden G., Fidyk W., Chmielik E., Pazdzior M., Grajek M., Kozik V., Sochanik A. (2021). Myxoma Virus Expressing LIGHT (TNFSF14) Pre-Loaded into Adipose-Derived Mesenchymal Stem Cells Is Effective Treatment for Murine Pancreatic Adenocarcinoma. Cancers.

[85] Babaei A., Bannazadeh Baghi H., Nezhadi A., Jamalpoor Z. (2021). In Vitro Anti-cancer Activity of Adipose-Derived Mesenchymal Stem Cells Increased after Infection with Oncolytic Reovirus. Adv. Pharm. Bull..

[86] Jazowiecka-Rakus J., Pogoda-Mieszczak K., Rahman M.M., McFadden G., Sochanik A. (2024). Adipose-Derived Stem Cells as Carrier of Pro-Apoptotic Oncolytic Myxoma Virus: To Cross the Blood-Brain Barrier and Treat Murine Glioma. Int. J. Mol. Sci..

[87] Josiah D.T., Zhu D., Dreher F., Olson J., McFadden G., Caldas H. (2010). Adipose-derived stem cells as therapeutic delivery vehicles of an oncolytic virus for glioblastoma. Mol. Ther..

[88] Draganov D.D., Santidrian A.F., Minev I., Nguyen D., Kilinc M.O., Petrov I., Vyalkova A., Lander E., Berman M., Minev B. (2019). Delivery of oncolytic vaccinia virus by matched allogeneic stem cells overcomes critical innate and adaptive immune barriers. J. Transl. Med..

[89] Chen Y., Xiang L.X., Shao J.Z., Pan R.L., Wang Y.X., Dong X.J., Zhang G.R. (2010). Recruitment of endogenous bone marrow mesenchymal stem cells towards injured liver. J. Cell Mol. Med..

[90] Chapel A., Semont A., Francois S., Mouiseddine M., Thierry D. (2005). Human Mesenchymal Stem Cells (MSC) Home at Injured Sites after Local Irradiation and Contribute To Reduce Radiation-Induced Intestinal Lesion. Blood.

[91] Xuan X., Tian C., Zhao M., Sun Y., Huang C. (2021). Mesenchymal stem cells in cancer progression and anticancer therapeutic resistance. Cancer Cell Int..

[92] Teo G.S., Ankrum J.A., Martinelli R., Boetto S.E., Simms K., Sciuto T.E., Dvorak A.M., Karp J.M., Carman C.V. (2012). Mesenchymal stem cells transmigrate between and directly through tumor necrosis factor-α-activated endothelial cells via both leukocyte-like and novel mechanisms. Stem Cells.

[93] Ponte A.L., Marais E., Gallay N., Langonné A., Delorme B., Hérault O., Charbord P., Domenech J. (2007). The in vitro migration capacity of human bone marrow mesenchymal stem cells: Comparison of chemokine and growth factor chemotactic activities. Stem Cells.

[94] Ullah M., Liu D.D., Thakor A.S. (2019). Mesenchymal Stromal Cell Homing: Mechanisms and Strategies for Improvement. iScience.

[95] Ge J., Guo L., Wang S., Zhang Y., Cai T., Zhao R.C., Wu Y. (2014). The size of mesenchymal stem cells is a significant cause of vascular obstructions and stroke. Stem Cell Rev. Rep..

[96] Prinyakupt J., Pluempitiwiriyawej C. (2015). Segmentation of white blood cells and comparison of cell morphology by linear and naïve Bayes classifiers. Biomed. Eng. Online.

[97] Moll G., Le Blanc K. (2015). Engineering more efficient multipotent mesenchymal stromal (stem) cells for systemic delivery as cellular therapy. ISBT Sci. Ser..

[98] Gao J., Dennis J.E., Muzic R.F., Lundberg M., Caplan A.I. (2001). The dynamic in vivo distribution of bone marrow-derived mesenchymal stem cells after infusion. Cells Tissues Organs.

[99] Schrepfer S., Deuse T., Reichenspurner H., Fischbein M.P., Robbins R.C., Pelletier M.P. (2007). Stem cell transplantation: The lung barrier. Transpl. Proc..

[100] Lee E.S., Im H.J., Kim H.S., Youn H., Lee H.J., Kim S.U., Hwang D.W., Lee D.S. (2014). In vivo brain delivery of v-myc overproduced human neural stem cells via the intranasal pathway: Tumor characteristics in the lung of a nude mouse. Mol. Imaging.

[101] Li Z., Oganesyan D., Mooney R., Rong X., Christensen M.J., Shahmanyan D., Perrigue P.M., Benetatos J., Tsaturyan L., Aramburo S. (2016). L-MYC Expression Maintains Self-Renewal and Prolongs Multipotency of Primary Human Neural Stem Cells. Stem Cell Rep..

[102] Portnow J., Synold T.W., Badie B., Tirughana R., Lacey S.F., D’Apuzzo M., Metz M.Z., Najbauer J., Bedell V., Vo T. (2017). Neural Stem Cell-Based Anticancer Gene Therapy: A First-in-Human Study in Recurrent High-Grade Glioma Patients. Clin. Cancer Res..

[103] Thaci B., Ahmed A.U., Ulasov I.V., Tobias A.L., Han Y., Aboody K.S., Lesniak M.S. (2012). Pharmacokinetic study of neural stem cell-based cell carrier for oncolytic virotherapy: Targeted delivery of the therapeutic payload in an orthotopic brain tumor model. Cancer Gene Ther..

[104] Ahmed A.U., Thaci B., Alexiades N.G., Han Y., Qian S., Liu F., Balyasnikova I.V., Ulasov I.Y., Aboody K.S., Lesniak M.S. (2011). Neural stem cell-based cell carriers enhance therapeutic efficacy of an oncolytic adenovirus in an orthotopic mouse model of human glioblastoma. Mol. Ther..

[105] Morshed R.A., Gutova M., Juliano J., Barish M.E., Hawkins-Daarud A., Oganesyan D., Vazgen K., Yang T., Annala A., Ahmed A.U. (2015). Analysis of glioblastoma tumor coverage by oncolytic virus-loaded neural stem cells using MRI-based tracking and histological reconstruction. Cancer Gene Ther..

[106] Fares J., Ahmed A.U., Ulasov I.V., Sonabend A.M., Miska J., Lee-Chang C., Balyasnikova I.V., Chandler J.P., Portnow J., Tate M.C. (2021). Neural stem cell delivery of an oncolytic adenovirus in newly diagnosed malignant glioma: A first-in-human, phase 1, dose-escalation trial. Lancet Oncol..

[107] Mooney R., Majid A.A., Batalla-Covello J., Machado D., Liu X., Gonzaga J., Tirughana R., Hammad M., Lesniak M.S., Curiel D.T. (2019). Enhanced Delivery of Oncolytic Adenovirus by Neural Stem Cells for Treatment of Metastatic Ovarian Cancer. Mol. Ther. Oncolytics.

[108] Hammad M., Cornejo Y.R., Batalla-Covello J., Majid A.A., Burke C., Liu Z., Yuan Y.C., Li M., Dellinger T.H., Lu J. (2020). Neural Stem Cells Improve the Delivery of Oncolytic Chimeric Orthopoxvirus in a Metastatic Ovarian Cancer Model. Mol. Ther. Oncolytics.

[109] Cornejo Y., Li M., Dellinger T.H., Mooney R., Rahman M.M., McFadden G., Aboody K.S., Hammad M. (2020). NSCs are permissive to oncolytic Myxoma virus and provide a delivery method for targeted ovarian cancer therapy. Oncotarget.

[110] Glass R., Synowitz M., Kronenberg G., Walzlein J.H., Markovic D.S., Wang L.P., Gast D., Kiwit J., Kempermann G., Kettenmann H. (2005). Glioblastoma-induced attraction of endogenous neural precursor cells is associated with improved survival. J. Neurosci..

[111] Aboody K.S., Brown A., Rainov N.G., Bower K.A., Liu S., Yang W., Small J.E., Herrlinger U., Ourednik V., Black P.M. (2000). Neural stem cells display extensive tropism for pathology in adult brain: Evidence from intracranial gliomas. Proc. Natl. Acad. Sci. USA.

[112] Imitola J., Raddassi K., Park K.I., Mueller F.J., Nieto M., Teng Y.D., Frenkel D., Li J., Sidman R.L., Walsh C.A. (2004). Directed migration of neural stem cells to sites of CNS injury by the stromal cell-derived factor 1alpha/CXC chemokine receptor 4 pathway. Proc. Natl. Acad. Sci. USA.

[113] Dey M., Yu D., Kanojia D., Li G., Sukhanova M., Spencer D.A., Pituch K.C., Zhang L., Han Y., Ahmed A.U. (2016). Intranasal Oncolytic Virotherapy with CXCR4-Enhanced Stem Cells Extends Survival in Mouse Model of Glioma. Stem Cell Rep..

[114] Aboody K.S., Bush R.A., Garcia E., Metz M.Z., Najbauer J., Justus K.A., Phelps D.A., Remack J.S., Yoon K.J., Gillespie S. (2006). Development of a tumor-selective approach to treat metastatic cancer. PLoS ONE.

[115] Danks M.K., Yoon K.J., Bush R.A., Remack J.S., Wierdl M., Tsurkan L., Kim S.U., Garcia E., Metz M.Z., Najbauer J. (2007). Tumor-targeted enzyme/prodrug therapy mediates long-term disease-free survival of mice bearing disseminated neuroblastoma. Cancer Res..

[116] Frank R.T., Edmiston M., Kendall S.E., Najbauer J., Cheung C.W., Kassa T., Metz M.Z., Kim S.U., Glackin C.A., Wu A.M. (2009). Neural stem cells as a novel platform for tumor-specific delivery of therapeutic antibodies. PLoS ONE.

[117] Cao P., Mooney R., Tirughana R., Abidi W., Aramburo S., Flores L., Gilchrist M., Nwokafor U., Haber T., Tiet P. (2017). Intraperitoneal Administration of Neural Stem Cell-Nanoparticle Conjugates Targets Chemotherapy to Ovarian Tumors. Bioconjug Chem..

[118] Lan X., Sun Z., Chu C., Boltze J., Li S. (2019). Dental Pulp Stem Cells: An Attractive Alternative for Cell Therapy in Ischemic Stroke. Front. Neurol..

[119] Fischer U.M., Harting M.T., Jimenez F., Monzon-Posadas W.O., Xue H., Savitz S.I., Laine G.A., Cox C.S. (2009). Pulmonary passage is a major obstacle for intravenous stem cell delivery: The pulmonary first-pass effect. Stem Cells Dev..

[120] van Beem R.T., Verloop R.E., Kleijer M., Noort W.A., Loof N., Koolwijk P., van der Schoot C.E., van Hinsbergh V.W., Zwaginga J.J. (2009). Blood outgrowth endothelial cells from cord blood and peripheral blood: Angiogenesis-related characteristics in vitro. J. Thromb. Haemost..

[121] Lin R.Z., Moreno-Luna R., Muñoz-Hernandez R., Li D., Jaminet S.C., Greene A.K., Melero-Martin J.M. (2013). Human white adipose tissue vasculature contains endothelial colony-forming cells with robust in vivo vasculogenic potential. Angiogenesis.

[122] Alphonse R.S., Vadivel A., Zhong S., McConaghy S., Ohls R., Yoder M.C., Thébaud B. (2015). The isolation and culture of endothelial colony-forming cells from human and rat lungs. Nat. Protoc..

[123] Ng C.Y., Cheung C. (2024). Origins and functional differences of blood endothelial cells. Semin. Cell Dev. Biol..

[124] Liu Y., Lyons C.J., Ayu C., O’Brien T. (2024). Recent advances in endothelial colony-forming cells: From the transcriptomic perspective. J. Transl. Med..

[125] Patel M.R., Jacobson B.A., Ji Y., Hebbel R.P., Kratzke R.A. (2020). Blood Outgrowth Endothelial Cells as a Cellular Carrier for Oncolytic Vesicular Stomatitis Virus Expressing Interferon-β in Preclinical Models of Non-Small Cell Lung Cancer. Transl. Oncol..

[126] Pagan J., Przybyla B., Jamshidi-Parsian A., Gupta K., Griffin R.J. (2013). Blood outgrowth endothelial cells increase tumor growth rates and modify tumor physiology: Relevance for therapeutic targeting. Cancers.

[127] Dudek A.Z., Bodempudi V., Welsh B.W., Jasinski P., Griffin R.J., Milbauer L., Hebbel R.P. (2007). Systemic inhibition of tumour angiogenesis by endothelial cell-based gene therapy. Br. J. Cancer.

[128] Zheng H., Fu G., Dai T., Huang H. (2007). Migration of endothelial progenitor cells mediated by stromal cell-derived factor-1alpha/CXCR4 via PI3K/Akt/eNOS signal transduction pathway. J. Cardiovasc. Pharmacol..

[129] Asahara T., Takahashi T., Masuda H., Kalka C., Chen D., Iwaguro H., Inai Y., Silver M., Isner J.M. (1999). VEGF contributes to postnatal neovascularization by mobilizing bone marrow-derived endothelial progenitor cells. Embo J..

[130] Raykov Z., Balboni G., Aprahamian M., Rommelaere J. (2004). Carrier cell-mediated delivery of oncolytic parvoviruses for targeting metastases. Int. J. Cancer.

[131] Iankov I.D., Blechacz B., Liu C., Schmeckpeper J.D., Tarara J.E., Federspiel M.J., Caplice N., Russell S.J. (2007). Infected cell carriers: A new strategy for systemic delivery of oncolytic measles viruses in cancer virotherapy. Mol. Ther..

[132] Colombo M., Mirandola L., Platonova N., Apicella L., Berta D.G., Lancellotti M., Lazzari E., Cobos E., Chiriva-Internati M., Chiaramonte R. (2015). Notch signaling drives myeloma cells homing to the bone marrow by regulating the CXCR4/CXCL12 axis. Clin. Lymphoma Myeloma Leuk..

[133] Liu C., Russell S.J., Peng K.W. (2010). Systemic therapy of disseminated myeloma in passively immunized mice using measles virus-infected cell carriers. Mol. Ther..

[134] Munguia A., Ota T., Miest T., Russell S.J. (2008). Cell carriers to deliver oncolytic viruses to sites of myeloma tumor growth. Gene Ther..

[135] Podshivalova E.S., Semkina A.S., Kravchenko D.S., Frolova E.I., Chumakov S.P. (2021). Efficient delivery of oncolytic enterovirus by carrier cell line NK-92. Mol. Ther. Oncolytics.

[136] Arai S., Meagher R., Swearingen M., Myint H., Rich E., Martinson J., Klingemann H. (2008). Infusion of the allogeneic cell line NK-92 in patients with advanced renal cell cancer or melanoma: A phase I trial. Cytotherapy.

[137] Pfirschke C., Schirrmacher V. (2009). Cross-infection of tumor cells by contact with T lymphocytes loaded with Newcastle disease virus. Int. J. Oncol..

[138] Cole C., Qiao J., Kottke T., Diaz R.M., Ahmed A., Sanchez-Perez L., Brunn G., Thompson J., Chester J., Vile R.G. (2005). Tumor-targeted, systemic delivery of therapeutic viral vectors using hitchhiking on antigen-specific T cells. Nat. Med..

[139] Kanzaki A., Kasuya H., Yamamura K., Sahin T.T., Nomura N., Shikano T., Shirota T., Tan G., Fukuda S., Misawa M. (2012). Antitumor efficacy of oncolytic herpes simplex virus adsorbed onto antigen-specific lymphocytes. Cancer Gene Ther..

[140] Thorne S.H., Negrin R.S., Contag C.H. (2006). Synergistic antitumor effects of immune cell-viral biotherapy. Science.

[141] Ilett E.J., Barcena M., Errington-Mais F., Griffin S., Harrington K.J., Pandha H.S., Coffey M., Selby P.J., Limpens R.W., Mommaas M. (2011). Internalization of oncolytic reovirus by human dendritic cell carriers protects the virus from neutralization. Clin. Cancer Res..

[142] Qiao J., Wang H., Kottke T., Diaz R.M., Willmon C., Hudacek A., Thompson J., Parato K., Bell J., Naik J. (2008). Loading of oncolytic vesicular stomatitis virus onto antigen-specific T cells enhances the efficacy of adoptive T-cell therapy of tumors. Gene Ther..

[143] Zhang Z., Yang N., Xu L., Lu H., Chen Y., Wang Z., Lu Q., Zhong K., Zhu Z., Wang G. (2024). Systemic delivery of oncolytic herpes virus using CAR-T cells enhances targeting of antitumor immuno-virotherapy. Cancer Immunol. Immunother..

[144] Joyce J.A., Fearon D.T. (2015). T cell exclusion, immune privilege, and the tumor microenvironment. Science.

[145] Mortezaee K., Majidpoor J. (2023). Mechanisms of CD8(+) T cell exclusion and dysfunction in cancer resistance to anti-PD-(L)1. Biomed. Pharmacother. Biomed. Pharmacother..

[146] Meng Y., Yu Z., Wu Y., Du T., Chen S., Meng F., Su N., Ma Y., Li X., Sun S. (2017). Cell-based immunotherapy with cytokine-induced killer (CIK) cells: From preparation and testing to clinical application. Hum. Vaccin. Immunother..

[147] Liu C., Suksanpaisan L., Chen Y.W., Russell S.J., Peng K.W. (2013). Enhancing cytokine-induced killer cell therapy of multiple myeloma. Exp. Hematol..

[148] Nikitina E., Larionova I., Choinzonov E., Kzhyshkowska J. (2018). Monocytes and Macrophages as Viral Targets and Reservoirs. Int. J. Mol. Sci..

[149] Reale A., Krutzke L., Cadamuro M., Vitiello A., von Einem J., Kochanek S., Palù G., Parolin C., Calistri A. (2023). Human Monocytes Are Suitable Carriers for the Delivery of Oncolytic Herpes Simplex Virus Type 1 In Vitro and in a Chicken Embryo Chorioallantoic Membrane Model of Cancer. Int. J. Mol. Sci..

[150] Iscaro A., Jones C., Forbes N., Mughal A., Howard F.N., Janabi H.A., Demiral S., Perrie Y., Essand M., Weglarz A. (2022). Targeting circulating monocytes with CCL2-loaded liposomes armed with an oncolytic adenovirus. Nanomedicine.

[151] Peng K.W., Dogan A., Vrana J., Liu C., Ong H.T., Kumar S., Dispenzieri A., Dietz A.B., Russell S.J. (2009). Tumor-associated macrophages infiltrate plasmacytomas and can serve as cell carriers for oncolytic measles virotherapy of disseminated myeloma. Am. J. Hematol..

[152] Muthana M., Rodrigues S., Chen Y.Y., Welford A., Hughes R., Tazzyman S., Essand M., Morrow F., Lewis C.E. (2013). Macrophage delivery of an oncolytic virus abolishes tumor regrowth and metastasis after chemotherapy or irradiation. Cancer Res..

[153] Kwan A., Nutter Howard F., Winder N., Atkinson E., Jailani A., Patel P., Allen R., Ottewell P., Shaw G., Conner J. (2022). Macrophage Delivered HSV1716 Is Active against Triple Negative Breast Cancer. Future Pharmacol..

[154] Combes F., Mc Cafferty S., Meyer E., Sanders N.N. (2018). Off-Target and Tumor-Specific Accumulation of Monocytes, Macrophages and Myeloid-Derived Suppressor Cells after Systemic Injection. Neoplasia.

[155] Franklin R.A., Liao W., Sarkar A., Kim M.V., Bivona M.R., Liu K., Pamer E.G., Li M.O. (2014). The cellular and molecular origin of tumor-associated macrophages. Science.

[156] McClellan J.L., Davis J.M., Steiner J.L., Enos R.T., Jung S.H., Carson J.A., Pena M.M., Carnevale K.A., Berger F.G., Murphy E.A. (2012). Linking tumor-associated macrophages, inflammation, and intestinal tumorigenesis: Role of MCP-1. Am. J. Physiol. Gastrointest. Liver Physiol..

[157] Olingy C.E., Dinh H.Q., Hedrick C.C. (2019). Monocyte heterogeneity and functions in cancer. J. Leukoc. Biol..

[158] Hourani T., Holden J.A., Li W., Lenzo J.C., Hadjigol S., O’Brien-Simpson N.M. (2021). Tumor Associated Macrophages: Origin, Recruitment, Phenotypic Diversity, and Targeting. Front. Oncol..

[159] Arwert E.N., Harney A.S., Entenberg D., Wang Y., Sahai E., Pollard J.W., Condeelis J.S. (2018). A Unidirectional Transition from Migratory to Perivascular Macrophage Is Required for Tumor Cell Intravasation. Cell Rep..

[160] Kranjc M.K., Novak M., Pestell R.G., Lah T.T. (2019). Cytokine CCL5 and receptor CCR5 axis in glioblastoma multiforme. Radiol. Oncol..

[161] Vanhaver C., van der Bruggen P., Bruger A.M. (2021). MDSC in Mice and Men: Mechanisms of Immunosuppression in Cancer. J. Clin. Med..

[162] Lang S., Bruderek K., Kaspar C., Höing B., Kanaan O., Dominas N., Hussain T., Droege F., Eyth C., Hadaschik B. (2018). Clinical Relevance and Suppressive Capacity of Human Myeloid-Derived Suppressor Cell Subsets. Clin. Cancer Res..

[163] Apodaca M.C., Wright A.E., Riggins A.M., Harris W.P., Yeung R.S., Yu L., Morishima C. (2019). Characterization of a whole blood assay for quantifying myeloid-derived suppressor cells. J. Immunother. Cancer.

[164] Fleming V., Hu X., Weller C., Weber R., Groth C., Riester Z., Hüser L., Sun Q., Nagibin V., Kirschning C. (2019). Melanoma Extracellular Vesicles Generate Immunosuppressive Myeloid Cells by Upregulating PD-L1 via TLR4 Signaling. Cancer Res..

[165] Valenti R., Huber V., Iero M., Filipazzi P., Parmiani G., Rivoltini L. (2007). Tumor-released microvesicles as vehicles of immunosuppression. Cancer Res..

[166] Arkhypov I., Özbay Kurt F.G., Bitsch R., Novak D., Petrova V., Lasser S., Hielscher T., Groth C., Lepper A., Hu X. (2022). HSP90α induces immunosuppressive myeloid cells in melanoma via TLR4 signaling. J. Immunother. Cancer.

[167] Eisenstein S., Coakley B.A., Briley-Saebo K., Ma G., Chen H.M., Meseck M., Ward S., Divino C., Woo S., Chen S.H. (2013). Myeloid-derived suppressor cells as a vehicle for tumor-specific oncolytic viral therapy. Cancer Res..

[168] Hawila E., Razon H., Wildbaum G., Blattner C., Sapir Y., Shaked Y., Umansky V., Karin N. (2017). CCR5 Directs the Mobilization of CD11b(+)Gr1(+)Ly6C(low) Polymorphonuclear Myeloid Cells from the Bone Marrow to the Blood to Support Tumor Development. Cell Rep..

[169] Jeong J., Suh Y., Jung K. (2019). Context Drives Diversification of Monocytes and Neutrophils in Orchestrating the Tumor Microenvironment. Front. Immunol..

[170] Trellakis S., Bruderek K., Hütte J., Elian M., Hoffmann T.K., Lang S., Brandau S. (2013). Granulocytic myeloid-derived suppressor cells are cryosensitive and their frequency does not correlate with serum concentrations of colony-stimulating factors in head and neck cancer. Innate Immun..

[171] Cassetta L., Bruderek K., Skrzeczynska-Moncznik J., Osiecka O., Hu X., Rundgren I.M., Lin A., Santegoets K., Horzum U., Godinho-Santos A. (2020). Differential expansion of circulating human MDSC subsets in patients with cancer, infection and inflammation. J. Immunother. Cancer.

[172] Marigo I., Bosio E., Solito S., Mesa C., Fernandez A., Dolcetti L., Ugel S., Sonda N., Bicciato S., Falisi E. (2010). Tumor-induced tolerance and immune suppression depend on the C/EBPbeta transcription factor. Immunity.

[173] Solito S., Falisi E., Diaz-Montero C.M., Doni A., Pinton L., Rosato A., Francescato S., Basso G., Zanovello P., Onicescu G. (2011). A human promyelocytic-like population is responsible for the immune suppression mediated by myeloid-derived suppressor cells. Blood.

[174] Dauer M., Schad K., Herten J., Junkmann J., Bauer C., Kiefl R., Endres S., Eigler A. (2005). FastDC derived from human monocytes within 48 h effectively prime tumor antigen-specific cytotoxic T cells. J. Immunol. Methods.

[175] Iankov I.D., Msaouel P., Allen C., Federspiel M.J., Bulur P.A., Dietz A.B., Gastineau D., Ikeda Y., Ingle J.N., Russell S.J. (2009). Demonstration of anti-tumor activity of oncolytic measles virus strains in a malignant pleural effusion breast cancer model. Breast Cancer Res. Treat..

[176] Li Z.L., Liang X., Li H.C., Wang Z.M., Chong T. (2016). Dendritic cells serve as a “Trojan horse” for oncolytic adenovirus delivery in the treatment of mouse prostate cancer. Acta Pharmacol. Sin..

[177] Na Y.R., Jung D., Gu G.J., Seok S.H. (2016). GM-CSF Grown Bone Marrow Derived Cells Are Composed of Phenotypically Different Dendritic Cells and Macrophages. Mol. Cells.

[178] Brasel K., De Smedt T., Smith J.L., Maliszewski C.R. (2000). Generation of murine dendritic cells from flt3-ligand-supplemented bone marrow cultures. Blood.

[179] In H., Park J.S., Shin H.S., Ryu S.H., Sohn M., Choi W., Park S., Hwang S., Park J., Che L. (2023). Identification of dendritic cell precursor from the CD11c(+) cells expressing high levels of MHC class II molecules in the culture of bone marrow with FLT3 ligand. Front. Immunol..

[180] Kim M.K., Kim J. (2019). Properties of immature and mature dendritic cells: Phenotype, morphology, phagocytosis, and migration. RSC Adv..

[181] Crespo H.J., Lau J.T., Videira P.A. (2013). Dendritic cells: A spot on sialic Acid. Front. Immunol..

[182] Zhu K., Shen Q., Ulrich M., Zheng M. (2000). Human monocyte-derived dendritic cells expressing both chemotactic cytokines IL-8, MCP-1, RANTES and their receptors, and their selective migration to these chemokines. Chin. Med. J..

[183] Ritter U., Wiede F., Mielenz D., Kiafard Z., Zwirner J., Körner H. (2004). Analysis of the CCR7 expression on murine bone marrow-derived and spleen dendritic cells. J. Leukoc. Biol..

[184] Creusot R.J., Yaghoubi S.S., Chang P., Chia J., Contag C.H., Gambhir S.S., Fathman C.G. (2009). Lymphoid-tissue-specific homing of bone-marrow-derived dendritic cells. Blood.

[185] Collin M., Bigley V. (2018). Human dendritic cell subsets: An update. Immunology.

[186] Scandella E., Men Y., Gillessen S., Förster R., Groettrup M. (2002). Prostaglandin E2 is a key factor for CCR7 surface expression and migration of monocyte-derived dendritic cells. Blood.

[187] Le Nouën C., Hillyer P., Winter C.C., McCarty T., Rabin R.L., Collins P.L., Buchholz U.J. (2011). Low CCR7-mediated migration of human monocyte derived dendritic cells in response to human respiratory syncytial virus and human metapneumovirus. PLoS Pathog..

[188] Moran T.P., Nakano H., Kondilis-Mangum H.D., Wade P.A., Cook D.N. (2014). Epigenetic control of Ccr7 expression in distinct lineages of lung dendritic cells. J. Immunol..

[189] Langlet C., Tamoutounour S., Henri S., Luche H., Ardouin L., Grégoire C., Malissen B., Guilliams M. (2012). CD64 expression distinguishes monocyte-derived and conventional dendritic cells and reveals their distinct role during intramuscular immunization. J. Immunol..

[190] Nakano H., Burgents J.E., Nakano K., Whitehead G.S., Cheong C., Bortner C.D., Cook D.N. (2013). Migratory properties of pulmonary dendritic cells are determined by their developmental lineage. Mucosal Immunol..

[191] Nakano H., Lin K.L., Yanagita M., Charbonneau C., Cook D.N., Kakiuchi T., Gunn M.D. (2009). Blood-derived inflammatory dendritic cells in lymph nodes stimulate acute T helper type 1 immune responses. Nat. Immunol..

[192] Schmidt N.O., Dührsen L., Reitz M., Henze M., Sedlacik J., Riecken K., Fehse B., Westphal M. (2014). Repeated intranasal application of neural stem cell-mediated enzym/prodrug therapy using a novel HSV-thimidine kinase variant improves therapeutic efficiency in an intracranial glioblastoma model. Neuro Oncol..

[193] Li G., Bonamici N., Dey M., Lesniak M.S., Balyasnikova I.V. (2018). Intranasal delivery of stem cell-based therapies for the treatment of brain malignancies. Expert. Opin. Drug Deliv..

